# Synthesis and Deconstruction of Polyethylene-type Materials

**DOI:** 10.1021/acs.chemrev.3c00587

**Published:** 2024-02-26

**Authors:** Simon T. Schwab, Maximilian Baur, Taylor F. Nelson, Stefan Mecking

**Affiliations:** Chair of Chemical Materials Science, Department of Chemistry, University of Konstanz, Universitätsstraße 10, 78464 Konstanz, Germany

## Abstract

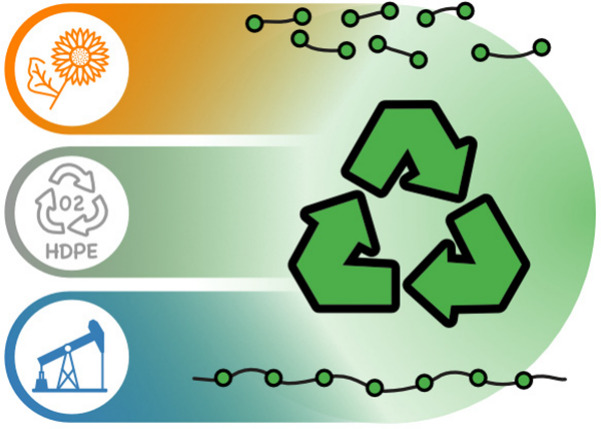

Polyethylene deconstruction to reusable smaller molecules is hindered by the chemical inertness of its hydrocarbon chains. Pyrolysis and related approaches commonly require high temperatures, are energy-intensive, and yield mixtures of multiple classes of compounds. Selective cleavage reactions under mild conditions (<ca. 200 °C) are key to improve the efficacy of chemical recycling and upcycling approaches. These can be enabled by introduction of low densities of predetermined breaking points in the polyethylene chains during the step-growth or chain-growth synthetic construction of designed-for-recycling polyethylene-type materials. Alternatively, they can be accomplished by postpolymerization functionalization of postconsumer polyethylene waste via dehydrogenation and follow-up reactions or through oxidation to long-chain dicarboxylates. Deconstruction of litter under environmental conditions via the aforementioned break points can alleviate plastics’ persistency, as a backstop to closed-loop recycling.

## 1 Introduction

Plastics are a key component of virtually any technology today. A myriad of applications in, e.g., the fields of construction, transportation, communication, water supply, or health care rely on the specific performance of polymer materials. The demand for plastic materials is ever increasing due to technological advances and an increasing world population with its desire for a high quality of life.^1^ This consumes enormous feedstock resources, as plastics are hardly reused after their useful service life. Currently, a large part of the plastic waste generated is placed in landfills, and a substantial portion is released to the environment in an uncontrolled way.^2^ Incineration with energy recovery essentially is a method of waste disposal, recovering a part of the caloric value of the plastic at the most.^3,4^ While the handling and legislation concerning plastic waste varies strongly between countries, a true recycling in the sense of a circular economy remains to be established anywhere. A closed loop that recycles polymers into an identical or similar-value application as in the preceding life cycle today is reality for only a small fraction of the plastic waste collected in advanced waste management systems.^5^ While such a truly circular approach will not be applicable and sensible for all types of waste streams, it is also evident that plastic waste represents a valuable resource that is not used efficiently today and overall plastics technology and economy could be designed in a more resource-efficient manner.^6^

Polyethylene is the largest produced synthetic polymer, with an annual production of more than 100 million tons. Polyethylene alone accounts for roughly one-third of the overall plastic production.^2^ Compared to other plastics, the emission of greenhouse gases per mass of polyethylene produced is comparatively low. Still, an entire cradle-to-grave life cycle, starting from crude oil and ending with incineration with energy recovery, produces 4.4 kg of CO_2_ equivalents per kg of polyethylene.^7^ The different types of polyethylene vary in their microstructure as a result of the production process, that is, catalytic insertion or free-radical chain growth, respectively, and the presence or absence of comonomers. High-density polyethylene (HDPE), low-density polyethylene (LDPE), and linear low-density polyethylene (LLDPE) each come in numerous different grades. Further variants comprise waxes and ultrahigh molecular weight polyethylene (UHMWPE), smaller in volume but still amounting to production volumes of millions of tons.^8,9^ Applications of polyethylenes range from food packaging films, detergent or milk bottles, mulch films, textiles, and high-performance fibers and ropes to fresh water pipes and orthopedic bearings to name only a few examples. Consequently, service life cycles range from short-term use (e.g., LDPE single-use packaging) to >50 years (e.g., HDPE pipes).

**Figure 1:**
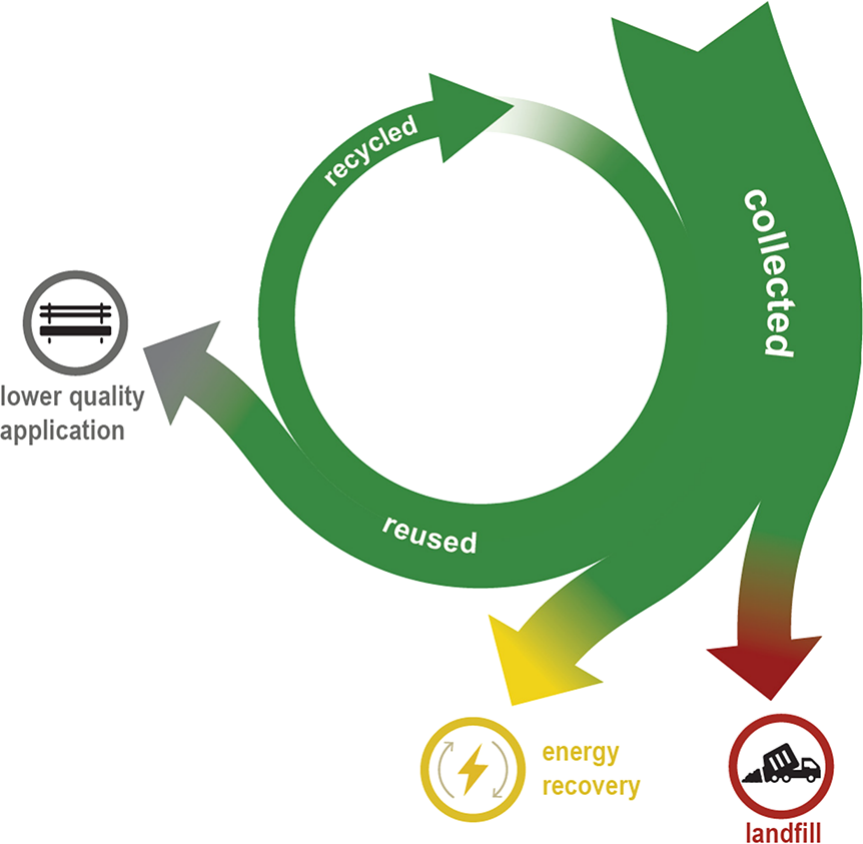
Fate of collected plastic waste in Western Europe.^2^

**Figure 2:**
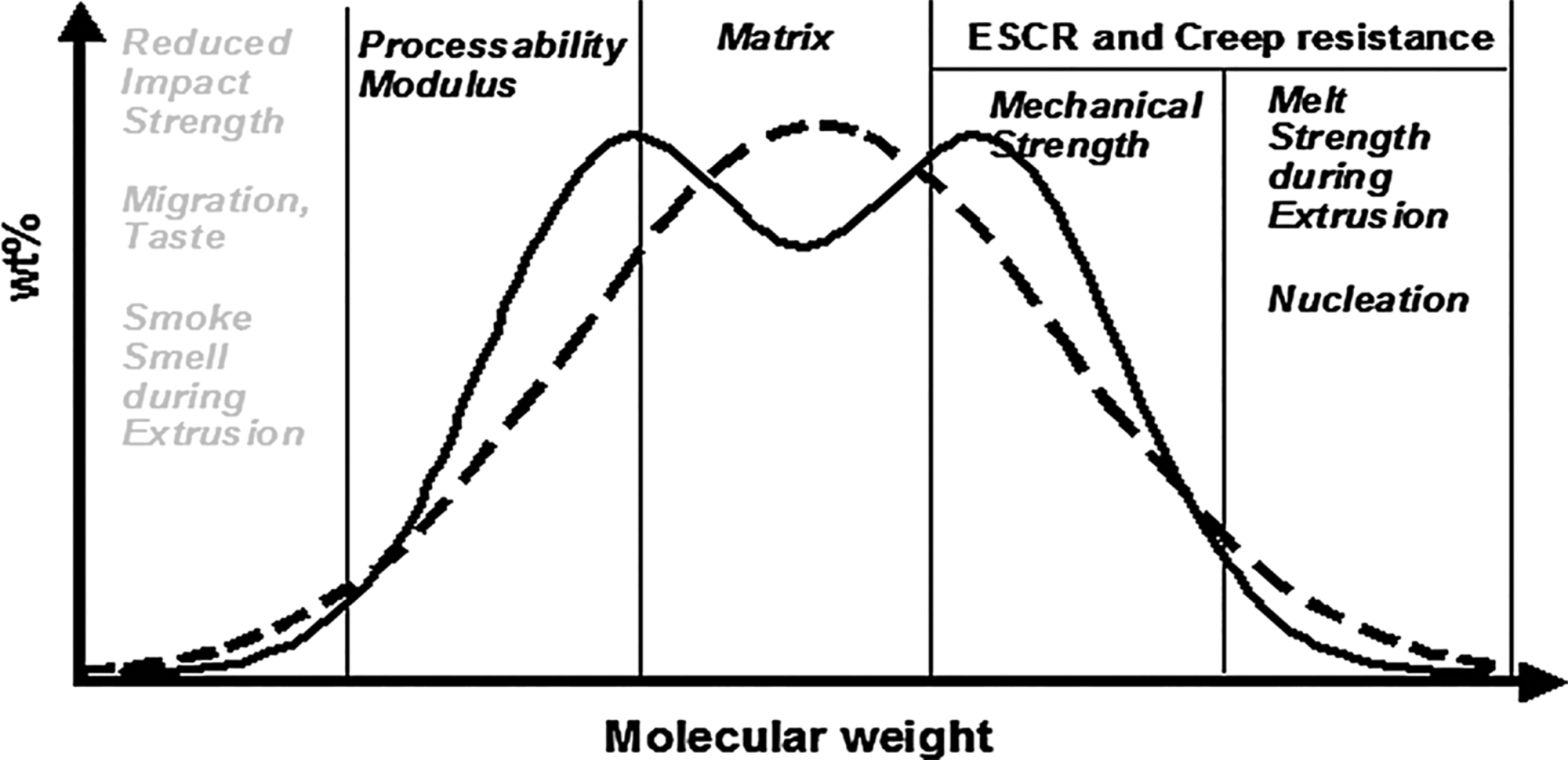
Schematic representation of a bimodal molecular weight distribution of polyethylene, and role of different molecular weight regimes in processing and materials properties (Reprinted with permission from *Tailor-Made Polymers Via Immobilization of Alpha-Olefin Polymerization Catalyst***2008**, 1–42. Copyright © 2008, Wiley-VCH).^12^

The mechanical recycling, requiring material that is washed and sorted in single-polymer streams, of PE is comparatively well-established vs most other plastics (with the exception of poly(ethylene terephthalate), PET). Yet, a closed-loop recycling to produce material suited for the same application is hardly practiced for collected polymer waste (Figure 1).^10,11^ One reason is that repeated processing and use lead to a deterioration of polymer properties. Further, the existence of many different grades complicates mechanical recycling. For example, PE molecular weight distributions are quite specifically defined for given processing methods and applications, with different molecular weight fractions serving different essential functions (Figure 2). Further, different PE products contain different stabilizers, processing aids, etc., in variable amounts.^12^ To achieve a sustainable plastic economy within the planetary boundaries, additional chemical recycling processes of existing polymers need to be established, and polymers specifically designed to enable such recycling are necessary.^10,13−15^

**Figure 3:**
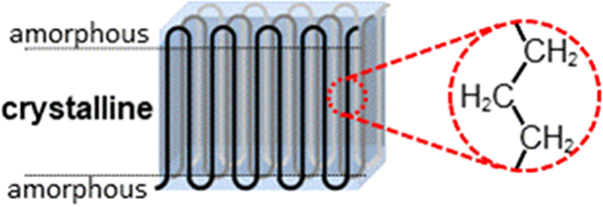
Polyethylene crystallinity arises from van der Waals interactions between adjacent stretched hydrocarbon segments, illustrated here for a folded chain crystallite.

The mechanical strength of polyethylene arises from crystalline order due to noncovalent (van der Waals) interactions between adjacent segments in all-trans conformation of hydrocarbon chains (Figure 3). This is particularly pronounced for HDPE, as it is composed of linear chains devoid of branches that would disturb this crystalline packing.^9^

**Figure 4:**
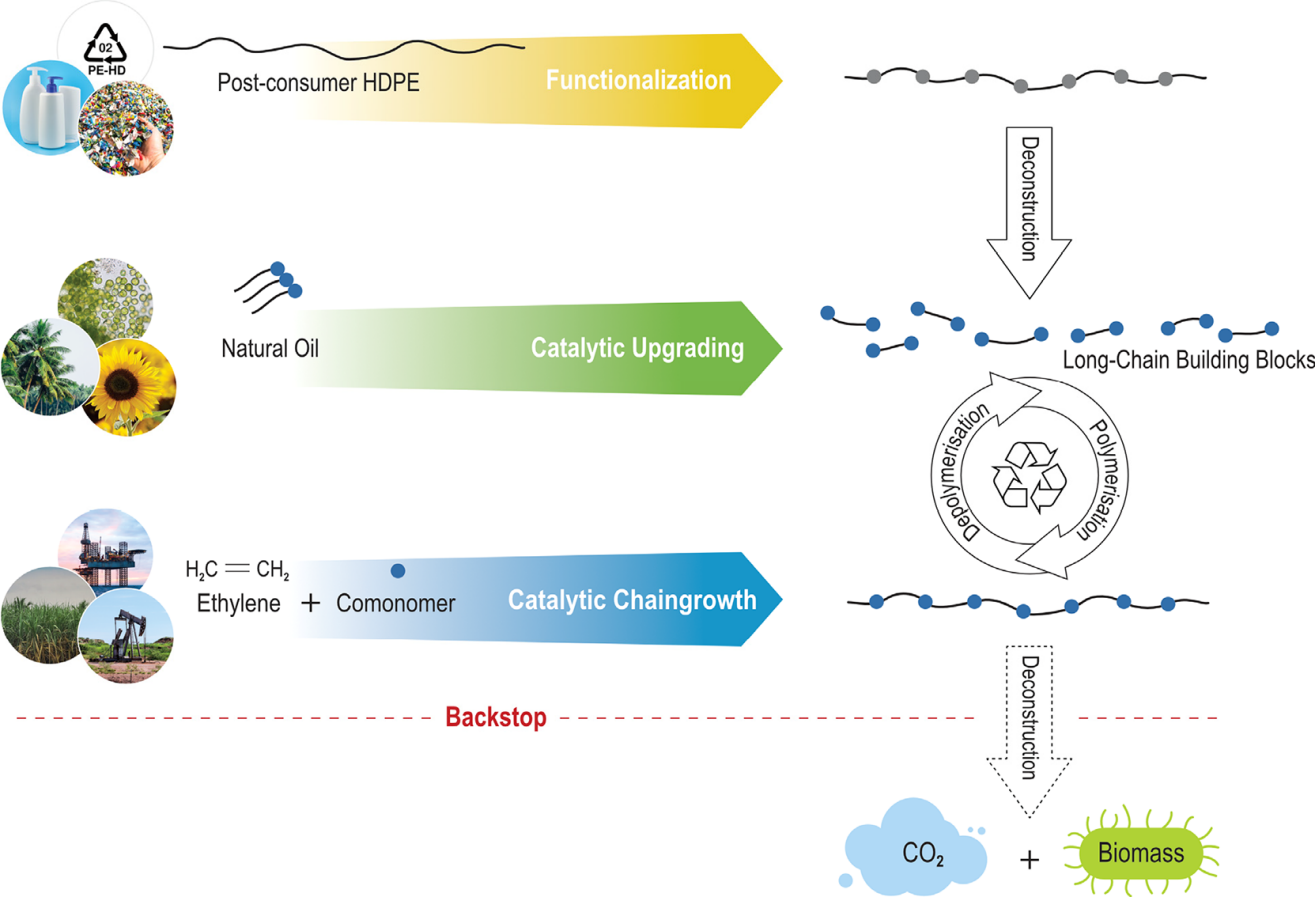
Access to PE-type materials from polyethylene waste, natural oil, and petroleum- or renewable-based ethylene. Chemical recycling is enabled via low densities of in-chain functional groups in a PE chain, and biodegradation acts as a backstop that prevents environmental accumulation of mismanaged plastic waste.

The chemically inert nature and C–C bond uniformity of the hydrocarbon chains hinders their breakdown back to the ethylene monomer. The formation of olefinic monomers from the saturated chains is kinetically hindered and thermodynamically disfavored.^16^ Thus, pyrolysis to liquid hydrocarbons and their steam cracking to generate olefins in both steps require high temperatures of up to 800 °C and are energy-consuming. Ethylene yields are limited in practice to around 10%.^4,17^ Catalytic cracking of polyethylene is of interest to reduce the temperatures required in particular (to around 300 °C).^18^ By contrast, the concepts for chemical recycling of PE-type polymers reviewed here operate at mild conditions (≤ca. 200 °C) and ideally avoid highly endothermic reactions (Figure 4).

Notably, this inert nature of polyethylene in conjunction with its hydrophobic, apolar, and crystalline nature also impedes degradation of material lost into the environment. Polyolefins can therefore persist for many decades or even centuries.^19^ While polymer deconstruction is considered here primarily within the context of recycling, the underlying principles of polymer design to enable such deconstruction also can facilitate the breakdown of plastic litter and prevent their accumulation; therefore, these complementary processes will also be discussed. The scope of “polyethylene-type” polymers is guided by the criterion of a hydrocarbon-dominated polymer crystallinity (cf. Figure 3) as a universal characteristic feature found in the various types of traditional polyethylenes. Typically, this is reflected in an orthorhombic crystal structure.

## 2 Synthesis

One approach to establish a circular plastics economy is the synthesis of polymers already tailored for deconstruction. Accordingly, functional groups as predetermined breaking points can be introduced into the polymer chain. A low density of such in-chain functional groups can enable different deconstruction pathways while not compromising the highly crystalline nature and desirable materials properties of PE. The inclusion of such predesigned break points can be achieved during chain-growth polymerization of olefins with functional comonomers. Alternatively, polyethylene-like chains with in-chain functional groups can also be generated by step-growth polycondensation reactions of long-chain difunctional monomers or by related ring-opening polymerizations of macrocycles.

### 2.1 Chain-Growth Polymers

Polyethylenes are produced industrially by catalytic (HDPE and LLDPE) or free-radical (LDPE) chain-growth polymerization. An inclusion of in-chain heteroatom-containing functional groups that could enable deconstruction has been a long-sought goal. However, traditional olefin polymerization catalysts are extremely sensitive to any polar reagents or impurities as a result of the high oxophilicity of their early transition-metal (d^0^) active sites.^20^ One approach to circumvent this limitation is the introduction of low densities of double bonds into polyolefin chains by copolymerization with, e.g., butadiene. Deconstruction via these double bonds commonly requires several reaction steps (cf. Chapter 3). A direct introduction of heteroatom-containing in-chain functional groups is enabled by recently discovered functional group tolerant catalysts or by free-radical polymerization, which will be discussed in more detail throughout this chapter.

Postpolymerization backbone oxidation reactions can also afford desired in-chain functional groups such as ketone or hydroxy groups. These postpolymerization reactions, however, can suffer from drawbacks such as uncontrolled chain cleavage and selectivity, if promoted in a free-radical fashion.^21,22^ Substantial efforts led to the development of more controlled, transition-metal catalyzed C–H functionalization.^23−27^ The introduction of keto and hydroxyl groups via this method can enhance materials properties, for example, enabling better adhesion to polar surfaces.^26^ Despite significant advances, the functional group selectivity remains a main challenge for postpolymerization modification of polyethylenes, and usually mixtures of ketones and hydroxyl groups are obtained from Ni- or Ru-catalyzed C–H functionalization.^25,26^ The synthesis of exclusively keto-containing polymers via this pathway was also reported but requires an additional oxidation step of such mixed “oxo-polyethylenes” by Cp*Ir-catalyzed transfer dehydrogenation using acetone as oxidant.^28^ Even though a high selectivity for keto groups (>99%) can be achieved initially with Cu catalysts and benzaldehyde as a reagent, the C–H oxidation results in a significant molecular weight decrease vs the original material.^27^ Thus, the oxidation of polyethylenes may unfold its potential rather in PE waste upcycling (cf. Section 3.2.2) or modification of materials properties than in the tailored synthesis of polymers designed for deconstruction.

**Figure 5:**
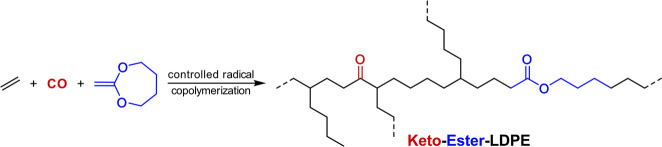
LDPE-like materials containing in-chain keto and ester groups from controlled free-radical terpolymerization of ethylene with CO and 2-methylene-1,3-dioxepane.^37^

The incorporation of carbon monoxide (CO) in high-pressure (∼1000 atm) free-radical ethylene polymerization to generate keto-modified LDPE was reported early on.^29−31^ Due to the relatively higher stability of the acyl radical formed after a CO-incorporation event, the chain-growth rate is decreased compared to ethylene homopolymerization.^30,32^ Like in ethylene homopolymerization, branched materials are obtained due to backbiting of the growing polyethylene radicals on the chain. In addition, branches adjacent to the carbonyl groups are prominent due to the propensity of H-abstraction from the α-methylene groups, −CH_2_C(=O)- (Figure 5).^32^ Recent laboratory-scale studies revealed that in reaction media that are inert to radical transfer reactions, namely, dimethyl carbonate^33^ or water, the (co)polymerization can be performed under comparatively moderate pressures of <350 atm to yield thermoplastic processable materials or film-forming aqueous dispersions, respectively.^32^ Notwithstanding the lowered growth rate by incorporation of CO, the copolymerization is practical for the industrial generation of LDPE materials. Keto-modified LDPEs with typically ca. 1 mol % of incorporated in-chain keto groups (*M*_n_ ∼ 5 × 10^4^ g mol^–1^; *Đ* ∼ 4) have been employed since the 1970s as photodegradable beverage six-pack slings, to enhance the degradation of littered material.^34−36^

In the context of recent studies of controlled free-radical copolymerization, the incorporation of keto as well as ester groups into polyethylene chains has been demonstrated.^37^ In the organometallic-mediated radical polymerization (OMRP) with cobalt(acetyl acetonate) complexes, degrees of branching are comparatively low due to the underlying reversible trapping of radicals (ca. 7/1000 carbon atoms). Notably, CO incorporations are much enhanced compared to reference uncontrolled copolymerizations. This is a result of the dual function of CO as comonomer and coordinating Lewis base. By terpolymerization with a cyclic ketene acetal, 2-methylene-1,3-dioxepane, lightly branched polyethylenes with in-chain keto as well as in-chain ester groups are accessible (∼2 mol % each, Figure 5).^37^ This is enabled by the propensity of the cyclic ketene acetal to undergo ring opening upon addition of a growing polymeryl radical to the double bond, to yield a new primary alkyl radical.^38^ The molecular weights reported so far (*M*_n_ ∼ 6 × 10^3^ g mol^–1^; *Đ* ∼ 2) call for improvement if applications as thermoplastics are targeted.^37^

In catalytic copolymerizations to linear polyethylenes, traditional early transition-metal catalysts are deactivated by carbon monoxide. Quenching by CO is in fact an established method to determine the number of active sites.^39^ Late transition-metal catalysts are not subject to such limitation, as evidenced by various large-scale industrial carbonylation processes for the synthesis of small molecules based on cobalt, nickel, rhodium, or iridium catalysts.^40^ In catalytic olefin-CO copolymerizations, the strong relative binding of CO and low barrier for CO insertion favors the formation of perfectly alternating polyketones.^41−47^ Alternating olefin-CO copolymers based on ethylene and ca. 5 mol % of propylene had been commercialized as high melting engineering thermoplastics (*T*_m_ ∼ 230 °C) by Shell under the trade name “Carilon”^48^ and have more recently been revived by Hyosung as “Poketone”.^49^ The polymerization process is based on cationic Pd(II) diphosphine complexes.^42−44^

**Figure 6:**
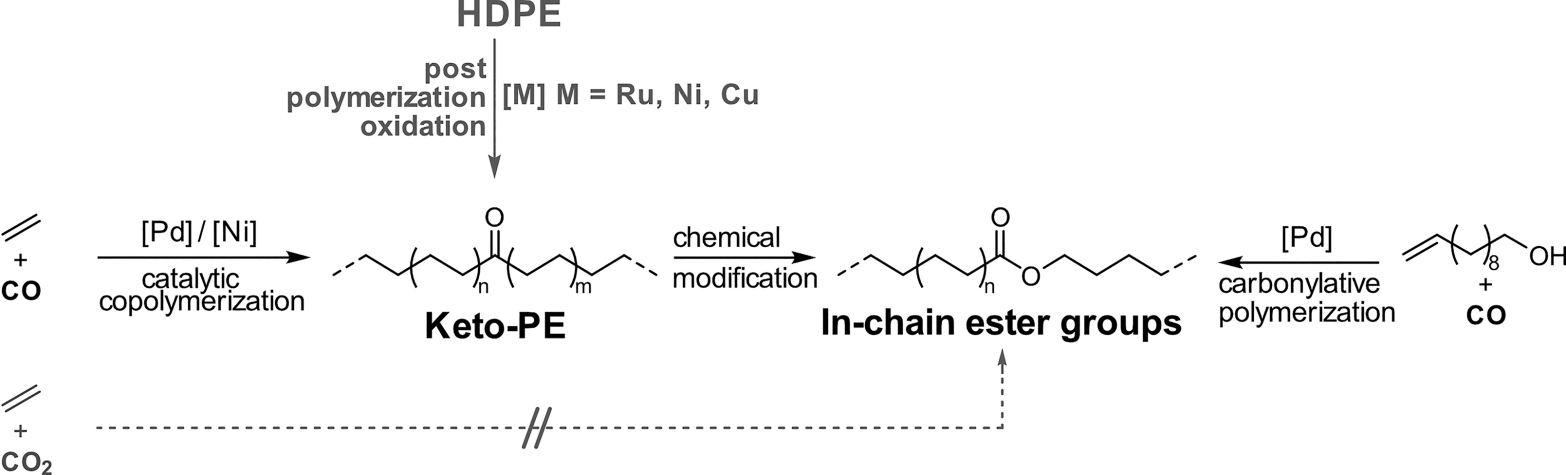
Routes to HDPE-like materials with in-chain keto and ester groups accessible by catalytic polymerizations. The postpolymerization oxidation route of polyethylene materials to similar materials is shown in comparison.

**Figure 7:**
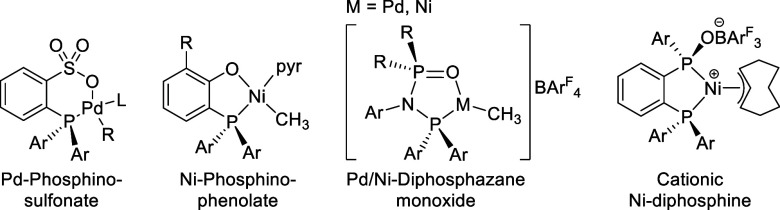
Reported catalysts for the nonalternating copolymerization of ethylene and CO.^52,60,66,69,70^

Overcoming the preference for CO incorporation is a key to enable catalytic polymerization to polyethylene-type materials with low densities of in-chain keto groups (Figure 6). Nonalternating ethylene CO copolymerizations are favored by a neutral charge of the catalyst that decreases the relative binding affinity of CO vs ethylene compared to cationic counterparts.^47,50−52^ Indeed, an inclusion of multiple ethylene-based repeat units in addition to alternating motifs was first observed for neutral Pd(II) phosphinosulfonate catalysts (Figure 7).^52^ Further studies reported materials with likely low keto incorporations; however, these were low molecular weight brittle polymers or oligomers which impeded studies of mechanical properties.^53^

Neutral Ni(II) phosphinophenolate catalysts are prominently employed in the Shell Higher Olefin process for ethylene oligomerization to linear 1-olefins. This is based on an effective competition of chain transfer with chain growth, with typical ratios of *v*_growth_ vs *v*_transfer_ of five to ten.^54−56^ Before this established picture, the finding of Shimizu et al. that bulky substituted phosphinophenolate catalysts polymerize ethylene to high molecular weight of *M*_n_ > 10^5^ g mol^–1^ was a breakthrough.^57,58^ This development was taken further with catalytic polymerizations in which chain transfer is virtually completely suppressed, resulting in a living character and the formation of ultrahigh molecular weights of *M*_n_ 3 × 10^6^ g mol^–1^, in the form of aqueous dispersions.^59^ Exposure of state-of-the-art catalysts to ethylene-CO mixtures with high olefin/CO ratios affords high molecular weight linear polyethylenes with exclusively nonalternating ketone motifs (“keto-PEs”), preferentially in the form of isolated carbonyls.^60−62^ Beneficially, the presence of CO increases molecular weights compared to ethylene homopolymerizations, likely due to a further suppression of β-hydride elimination (to *M*_n_ 2 × 10^5^ g mol^–1^; *M*_w_ 4 × 10^5^ g mol^–1^).^60^ At the target incorporations of ca. 0.3 to 3 mol % keto units these do not disturb the polyethylene crystalline structure, nor is the melting point impacted significantly^60^ as the incorporation of keto-groups into the crystalline lattice of polyethylene is associated with a rather low energy penalty.^63−65^

Consequently, these keto-PEs are on par with commercial HDPE concerning their tensile properties, as determined on injection-molded specimens. At the same time they are photodegradable (cf. section 3.1).^60^ For the synthesis of these materials, the dosing of high ethylene/CO ratios was achieved by premixing in a high-pressure syringe pump^60^ or by mass flow meters with a customized control system.^61^ Notably, both setups allow for the convenient switching between natural isotopic abundance CO and isotopically pure ^13^CO, the latter facilitating exhaustive microstructure analysis by ^13^C NMR spectroscopy analysis and also monitoring of photodegradation (cf. Chapter 3). In parallel studies, Nozaki et al. demonstrated the formation of high molecular weight (*M*_n_ 6 × 10^4^ g mol^–1^) linear polyethylenes with an excellent ratio of isolated in-chain carbonyls (99%) employing their (dimenthylphosphino)sulfonate Pd(II) catalyst. Metal carbonyls like [Fe(CO)_5_] were elegantly used as a source of the keto groups.^66^ As an alternative way of generating low concentrations of CO for nonalternating copolymerization, the electroreduction^67^ or photoreduction^68^ of carbon dioxide in a separate compartment of a polymerization reactor has also been reported.

Nonalternating ethylene-CO copolymerizations have also been demonstrated more recently with cationic Pd(II) catalysts but at still relatively high keto incorporations that also reflect in significantly higher melting points compared to polyethylene.^69,70^ Further development and studies of these catalysts, as well as their Ni(II) analogues, yielded keto-PEs with low CO incorporation and high molecular weights (*M*_n_ up to 10^5^ g mol^–1^).^71^

**Figure 8:**
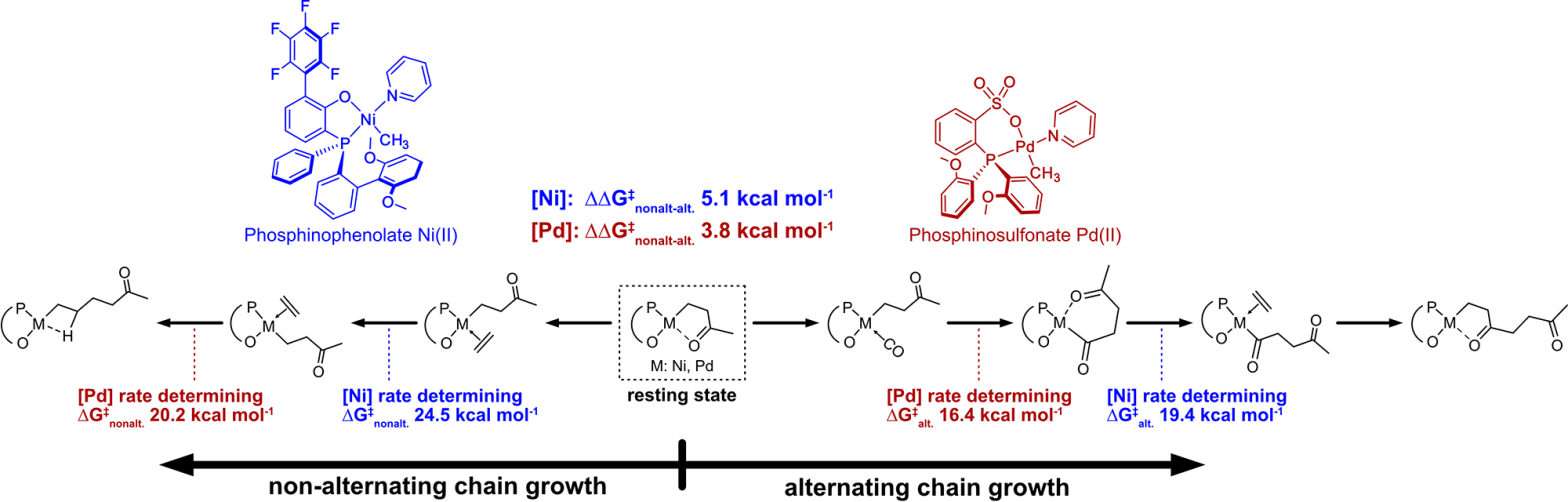
Mechanistic steps and activation barriers of nonalternating vs alternating ethylene/CO copolymerization for Pd-phosphinosulfonate (red) and Ni-phosphinophenolate catalysts (blue) as calculated by DFT.^62^

From a mechanistic point of view the ability of the aforementioned neutral catalysts to generate keto-PE arises from the barrier of the nonalternating pathway of chain growth being competitive to that of alternating chain growth (Figure 8).^45,46,62^ DFT studies starting from a five-membered chelate formed by CO insertion revealed that Ni-phosphinophenolate catalysts indeed have a ΔΔ*G*^‡^_alt-nonalt_ similar to Pd-phosphinosulfonate complexes despite differing in the nature of the rate-determining steps for both pathways.^62^ In the phosphinosulfonate Pd(II) system, the decisive steps are CO insertion for the alternating and ethylene insertion for the nonalternating pathway. The key steps in Ni-phosphinophenolate systems are ethylene coordination to open a six-membered chelate for the alternating, and metal–alkyl cis–trans isomerization for the nonalternating pathways. These pathways are further governed by different parameters: alternating chain growth is mostly influenced by the electronic properties of the catalyst and nonalternating chain growth by catalyst sterics, respectively.^62^

Despite conveniently introducing in-chain carbonyl groups and imparting photodegradability, the keto-PE materials obtained from ethylene/CO copolymerization are not inherently susceptible to direct chemical deconstruction by simple reactions, such as solvolysis of ester groups. Nevertheless, the in-chain keto groups can provide an array of potential chemical modifications^72−74^ and can serve as a platform to make polyolefins more prone for subsequent chemical deconstruction^75^ as demonstrated recently by Baeyer–Villiger oxidation of keto-PEs to polyethylenes containing a combination of in-chain keto and ester groups.^76^ While HDPE-like materials properties were retained in these keto-ester-PEs, the combination of functional groups allowed for photolytic degradation via the keto groups as well as chemical deconstruction of the ester groups by methanolysis.^76^

In-chain ester groups can theoretically be included via catalytic chain-growth copolymerization of carbon dioxide (CO_2_) and ethylene (Figure 6). The utilization of CO_2_ as a comonomer with olefins has long been discussed^77−80^ but suffers from thermodynamic and kinetic limitations. Thermodynamic calculations by Miller^77^ and DFT studies by Nozaki^78^ concluded that alternating ethylene/CO_2_ copolymerization is thermodynamically strongly disfavored. Nonalternating copolymerization with an excess ethylene incorporation (ethylene/CO_2_ > 2.4), however, was found to be, in principle, thermodynamically accessible,^77,80^ and a generic catalytic cycle following the coordination–insertion mechanism was proposed by Müller et al.^79^ Nevertheless, the substantially lower activation barrier for the alternative “escape route” via ethylene homopolymerization compared to CO_2_ copolymerization^78,79^ prevents incorporation of CO_2_ into the polymer chain, and effective kinetic pathways are yet to be found. In ethylene polymerizations by cationic Pd(II)^81^ or neutral Ni(II) salicylaldiminato catalysts^82−84^ in supercritical carbon dioxide (*sc*CO_2_) as a reaction medium, the *sc*CO_2_ was found to be entirely inert. In contrast, direct catalytic conversion of ethylene and other olefins with one equivalent of CO_2_ to acrylates is well-documented, but this is enabled by the formation of acrylic acid salts as a driving force.^85,86^ These significant, if not prohibitive, hurdles for a direct in-chain CO_2_ incorporation can be circumvented by reaction with 1,3-butadiene to an unstable lactone intermediate. Even though polymers containing up to 30% CO_2_ are formed, most of these polymers contain the ester groups not as main chain links, and they do not possess PE-like chain microstructures or properties.^78,80,87,88^ Only recently, Tonks and co-workers reported a well-defined polyester formed via a CO_2_-butadiene pathway.^89^ The materials obtained by ring-opening polymerization (ROP) of the formed lactone intermediate, however, possessed properties of an amorphous polyester (with *T*_g_ ∼ −40 °C) rather than resembling semicrystalline polyethylene-type materials.

**Figure 9:**
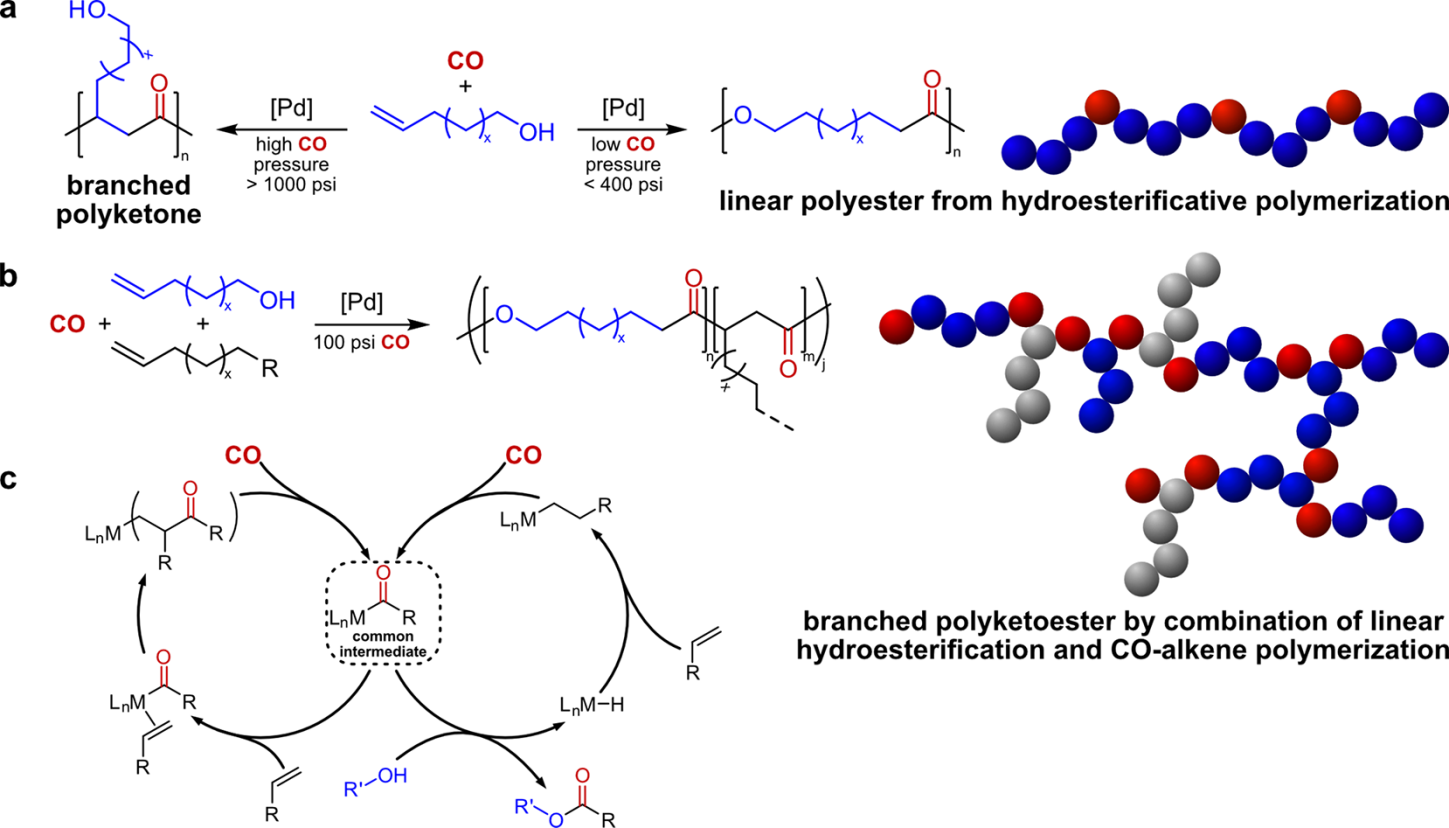
(a) Concept of hydroesterificative polymerization to linear polyesters^90,91^ and competitive alkene copolymerization.^94^ (b) Formation of branched polyketoesters by combining compatible pathways of linear hydroesterificative and carbonylative alkene polymerization.^96^ (c) Catalytic cycles of hydroesterificative and carbonylative alkene polymerization with common acyl intermediate, which allows for switching of the polymerization pathways (Figure 9c: Reprinted with permission from *ACS Catal*. **2022**, 12, 14629–14636. Copyright © 2022, American Chemical Society).^96^

Nevertheless, in addition to the aforementioned free-radical polymerization approach, catalytic approaches to directly incorporate in-chain ester groups in polyethylene chains during polymerization have also been reported. Hydroesterificative polymerization of a linear, long-chain ω-unsaturated alcohol with CO can yield a linear polyester (Figure 9a, right pathway) with *M*_n_ up to 1.7 × 10^4^ g mol^–1^ and *T*_m_ > 70 °C.^90,91^ Here, the formed cobalt- or palladium-acyl intermediate after insertion of CO is trapped by the alcohol group of the monomer to form the respective (poly)ester.^92^ Monomer extension of the ω-unsaturated alcohol by etherification expands the scope of accessible microstructures.^93^ Since hydroesterification and CO/alkene copolymerization^94^ share the same metal-acyl intermediate^95^ (Figure 9c), the pathways of hydroesterification and CO/alkene copolymerization can compete, if suitable cationic Pd-bisphosphine catalysts are chosen and a polyketoester with tunable ratios of in-chain ester and keto groups can be obtained (Figure 9b).^96^ As the formation of keto repeat units goes along with formation of a branch, branched microstructures are obtained: short-chain branches are formed by the nonfunctionalized comonomer (e.g., 1-hexene) and long-chain branches originate from the functional comonomer which is incorporated as keto repeat unit and then further reacted on the hydroxyl group. These branched copolymers exhibited only rather low molecular weights with high dispersities (*M*_n_ (1.5–25) × 10^3^ g mol^–1^, *Đ* 2.4–10.2) and low melting points *T*_m_ 13–57 °C.^96^

**Figure 10:**
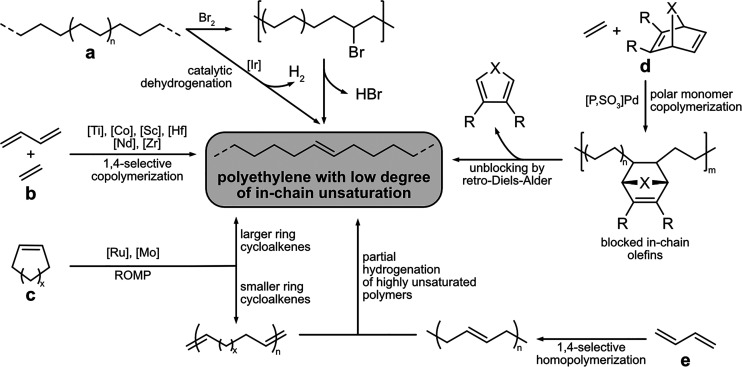
General overview of different approaches to polyethylene with a low degree of in-chain unsaturation via chain-growth polymerization and post-polymerization functionalization.

An alternative to provide in-chain groups that can eventually serve for deconstruction (cf. Chapter 3.2.1) is the introduction of unsaturation during polymer synthesis. This can be achieved by catalytic insertion copolymerization of butadiene, or ring-opening metathesis polymerization (ROMP) of cycloolefins (Figure 10, c). These all-hydrocarbon comonomers do not require catalysts tolerant toward heteroatom-containing monomers, and consequently also very oxophilic catalysts can be employed.

Incorporation of butadiene comonomer in a 1,4-fashion in catalytic copolymerization of ethylene^97−101^ (or propylene^102−105^) can yield in-chain double bonds, with the degree of unsaturation in the polyolefin chain being controlled by monomer ratios. Terpolymerization with other α-olefins can lead to unsaturated, LLDPE-like structures.^99,106,107^ A variety of catalysts have been reported for ethylene butadiene copolymerization.^100^ Most commonly, lanthanide catalysts based on Nd^97,106,108−110^ or Sc^101,109,111^ are used for copolymerization of ethylene and butadiene. But also group IV catalysts based on titanocenes,^98,112^ zirconocenes,^103−105,113,114^ or hafnium^102,113^ are suitable. Even cobalt-based late transition-metal catalysts were reported.^115^ However, the activity of most catalysts is hampered by butadiene, and other microstructure motifs commonly occur in addition to the desired in-chain olefins. Conventional Ziegler catalysts mostly yield alternating copolymers or multiblock copolymers^100,106^ and therefore are not ideal for the incorporation of a low density of unsaturation. The 1,2-insertion of butadiene results in side-chain olefins. Their *in situ* cyclization by reinsertion has been widely reported and is used in the synthesis of ethylene–butadiene elastomers.^100,106,116^ Selectivity for 1,4-insertion over 1,2-insertion is required for incorporation of a low density of in-chain unsaturation.

Several catalysts have been reported to yield good 1,4-selectivity while also enabling low amounts of butadiene in the polymer without forming block-like or alternating sequences under appropriate copolymerization conditions. These are usually: low butadiene feed-ratios, suitable activator species, and activator-catalyst ratio.^98,101−104,112,115,117^ Silica-supported CpTiCl_3_ activated with triisobutyl or trioctyl aluminum can yield ethylene–butadiene copolymers with random distribution of olefin groups between 0.5 and 2.5 mol % and high molecular weights of up to 1.1 × 10^5^ g mol^–1^.^98^ Postmetallocene single-site catalysts like [Me_2_Si(N^t^Bu)(Me_4_Cp)]TiCl_2_ can also give high molecular weight copolymers (∼3 × 10^5^ g mol^–1^) with adjustable degrees of in-chain unsaturation between 0.5 and 16 mol %. Especially at lower butadiene ratios, 1,4-insertion prevails (>70%) over 1,2-vinylic structures even at 16 mol % butadiene incorporation.^112^ Bis(arylimino)pyridyl cobalt(II) catalysts can yield ethylene-rich copolymers with prevailing 1,4-incorporation (70–97% vs 1,2) when activated with modified methylaluminoxane (MMAO), even though only low molecular weights (<1.4 × 10^4^ g mol^–1^) were obtained.^115^ Melting points of these copolymers with low degrees of in-chain unsaturation of around approximately 100–120 °C agree with the expected behavior of a polyethylene with occasional defects in the form of unsaturation within the chain.^98,112,115^ Further, tetradentate [OSSO]-bis(phenolato)Ti(IV)^117^ and half-sandwich thiophene-fused cyclopentadienyl Sc(III) catalysts^101^ have also been reported for ethylene–butadiene copolymerization with exclusively 1,4-selectivity but favored incorporation of butadiene in alternating blocks, thus lacking truly polyethylene-like material properties.^101,117^

In addition to ethylene as comonomer, the copolymerization of butadiene and propylene with good 1,4-selectivity and low degrees of in-chain unsaturation to semicrystalline polypropylene-like polymers with high molecular weights (>5 × 10^4^ g mol^–1^) and melting points >100 °C was reported for *ansa*-bis(indenyl)zirconocene catalysts^103−105^ and bridged biphenylphenoxide Hf(IV) catalysts.^102^

Recently, also the incorporation of “blocked” in-chain olefin groups by Pd-catalyzed copolymerization of ethylene with oxa-norbornadienes was reported. These can subsequently be unblocked by retro Diels–Alder cleavage upon heating in solution or polymer melt, resulting in unsaturated HDPE chains with up to 2.2 mol % in-chain olefins, while high molecular weights are obtained after unblocking (*M*_n_ ∼ 3 × 10^4^ g mol^–1^).^118^

Aliphatic polymers with in-chain double bonds can also be accessed by ROMP of cyclic monomers.^119−122^ However, the degree of unsaturation is usually high enough that the products do not resemble polyethylenes. For example, eight-membered ring cyclooctene monomers are efficiently polymerized by ROMP, to yield products with a double bond for every sixth methylene carbon of the chain.^123,124^ Larger ring-size monomers on the other hand are often not readily available and require multistep syntheses, and the driving force for their ROMP is limited.^121,123^ The high density of internal double bonds also leads to relatively short-chain monomers when deconstructed to α,ω-telechelics, as compared to the longer-chain α,ω-telechelics accessible from olefin-butadiene copolymers. Alternatively, an unsaturated polymer obtained from ROMP can be subjected to partial hydrogenation to reduce the degree of unsaturation and therefore better resemble thermal and solid-state properties of polyethylene, as demonstrated for the ROMP of cyclopentene.^125^ The obtained polymer could be partially hydrogenated by employing variable amounts of *p*-toluenesulfonyhydrazide to polyethylenes with tailored degrees of unsaturation.^125^ Similarly, partial hydrogenation of amorphous and highly unsaturated 1,4-polybutadiene homopolymer with [(Ph_3_P)_3_RhCl] can give polyethylene-like polymers with varying degrees of unsaturation between 2.6 and 16 mol %, with crystallinity and melting points strongly dependent on the amount of residual in-chain olefins (*T*_m_: 78–118 °C).^126^

**Figure 11:**
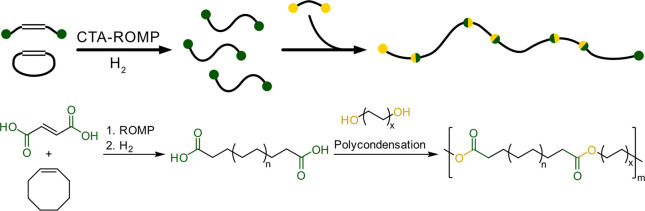
Synthesis concept of long-chain α,ω-carboxy telechelics from CTA-ROMP as PE-like building blocks.^132,135^

Another possibility arising from ROMP is the direct synthesis of long-chain α,ω-telechelic molecules. Instead of cleaving an unsaturated polymer by oxidation or metathesis, the ROMP is carried out in the presence of a functionalized acyclic alkene, which acts as chain transfer agent (CTA).^127^ An approach to access such α,ω-telechelics has been explored extensively by Grubbs and Hillmyer.^124,128−134^ Carboxyl-functionalized telechelic long-chain monomers can be precisely synthesized by employing an unsaturated diacid, such as maleic acid.^132^ For the synthesis of hydroxyl-terminated telechelic molecules, diacetyl alkenes are used as CTA in ROMP and converted to the free alcohol by hydrolysis.^129,131,133^ Recently, Hillmyer and co-workers reported the direct synthesis of a hydroxyl-terminated α,ω-telechelic molecule with 7-hexadecene-1,16-diol as CTA.^134^ Hydrogenation of the unsaturated α,ω-telechelic molecules leads to saturated PE-like segments,^132−134^ which can be used as long-chain monomers for polycondensation reactions to recyclable PE-like polymers. Particularly, long-chain α,ω-hydroxy or carboxy telechelic molecules offer potential in view of application as long-chain building blocks toward recyclable polyethylene-like polymers (Figure 11), as demonstrated for linear, HDPE-type^135,136^ as well as branched, therefore rather LLDPE-like building blocks.^136^

### 2.2 Step-Growth Polymers

An alternative to construction of polyethylene-type materials with in-chain functional groups by chain-growth copolymerizations is the synthesis of such materials via polymerization reactions of these very functional groups (Figure 4, right). An illustrative example is the A_2_ + B_2_ polyesterification of long-chain dicarboxylic acids, A-(CH_2_)_n_-A (A = carboxylate) with long-chain diols B-(CH_2_)_m_-B (B = hydroxyl group). This concept requires straightforward access to suitable long-chain monomers, which is provided by state-of-the-art catalytic conversions of suitable long-chain substrates, primarily unsaturated fatty acids.

#### 2.2.1 Access to Monomers

Fatty acids offer themselves as starting materials for the synthesis of long-chain difunctional monomers as they already provide long, linear methylene sequences endowed with a terminal carboxylate group.

**Figure 12:**
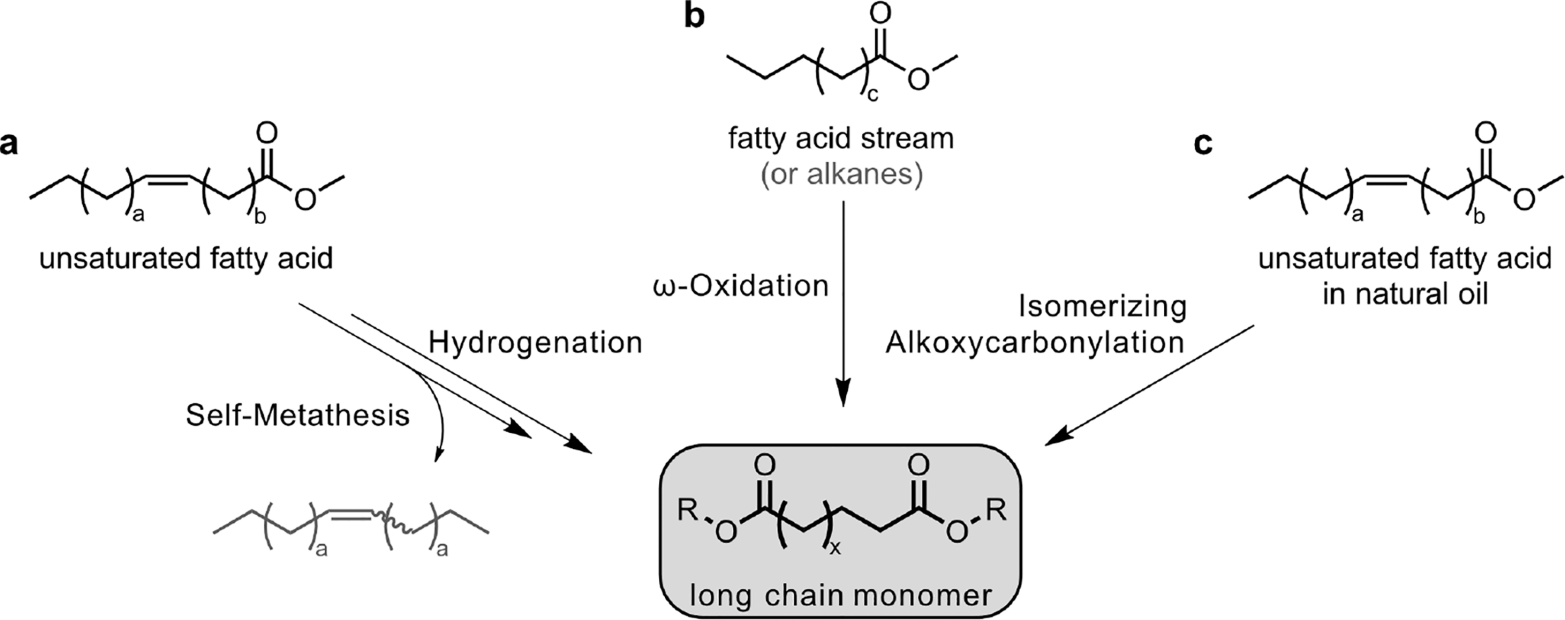
Schematic diagram for the synthesis of long-chain monomers from fatty acids via self-metathesis, isomerizing alkoxycarbonylation, and ω-oxidation.

Self-metathesis of unsaturated fatty acids or their ester analogs yields a stoichiometric mixture of internally unsaturated alkenes and α,ω-dicarboxylates (Figure 12a). Catalytic double-bond hydrogenation of the latter affords the target long-chain saturated dicarboxylate monomers, e.g., C_18_ octadecanedioate from oleate feedstocks or C_26_ from erucates. Molecular catalysts, like Grubbs II ruthenium alkylidene or molybdenum catalysts, are suitable for olefin metathesis of plant oil feedstocks including extensively produced crops like soy bean or palm oil to generate the α,ω-dicarboxylates.^137,138^ Elevance (now Wilmar) has commercialized olefin metathesis of fatty acids in a large-scale biorefinery in Indonesia since 2013, and offers C_18_ dicarboxylates commercially, in a quality suitable for polycondensation.^139,140^

Isomerizing alkoxycarbonylation (Figure 12c) of unsaturated fatty acid esters generates a second ester group, at the other terminus of the fatty acid substrate, from reaction of the internal double bond with carbon monoxide and methanol or another alcohol.^141,142^ In order to generate the new ester group not at the original double bond position in the center of the fatty acid chain but at the terminus, catalysts are required that “walk” back and forth along the fatty acid chain easily and selectively undergo ester formation only when at the terminal position.^143^ This can be achieved with Pd(II) catalysts with bulky substituted diphosphines like the commercially available 1,2-bis(di-*tert*-butylphosphino)xylene.^144,145^ By this kinetic control, selectivities up to 95% at virtually complete conversions have been achieved.^146^ Unlike olefin metathesis, the entire fatty acid substrate is incorporated in the product. As the second carbonyl group originates from the carbon monoxide substrate, odd-numbered products are formed, e.g., C_19_ from high oleic sunflower oil or C_23_ from erucic feedstock.^147^ Formates such as methyl formate can act as CO surrogates in this process to produce CO *in situ*, alleviating the need for a CO supply infrastructure.^148,149^ Isomerizing carbonylation has been demonstrated on a laboratory scale (up to ca. 0.3 kg), yielding long-chain diesters or dicarboxylic acids (from hydroxycarbonylation with water) in polycondensation-grade quality.^141^

The aforementioned reactions yield long-chain difunctional monomers of similar or equal chain length as the fatty acid substrate. Ultra long-chain difunctional linear products can be generated through “chain doubling” by combination of dynamic catalytic isomerizing crystallization with olefin metathesis. This yields, for example, a C_48_ linear diester as polyethylene-like telechelic from erucate.^150^

In addition to the aforementioned carboxylates, long-chain diols are also attractive monomers for the generation of polyethylene-type materials. They are accessible from the α,ω-diesters via reduction with metal hydrides or ruthenium-catalyzed reduction with molecular hydrogen.^141,151,152^

As an alternative to the catalytic upgrading of oils, biotechnological transformation of saturated or unsaturated fatty acids, or alkanes, allows for an oxidation of the terminal methyl carbon. The ω-oxidation of C_14_ to C_22_ fatty acids was enabled by the selective blocking of specific parts of the β-oxidation pathway of different yeast strains.^153^ The target monomers could be generated in concentrations of up to 160 g L^–1^ with high substrate conversion efficiency. The need for additional nutrients and a rather complex workup procedure of fermentation broths are limitations of this process. ω-Oxidation was developed in the 1990s by Henkel (now Emery Oleochemicals) to produce C_18_ diacid, among others, on a pilot scale.^154^ Today, Cathay Industrial Biotech^155^ also offers C_14_ and higher chain length dicarboxylic acids sourced from fermentation.

By blocking the β-oxidation pathway even further upstream, unsymmetrical ω-hydroxy fatty acids are also accessible.^156^ These can be polymerized via step-growth AB polycondensation to linear polyhydroxyalkanoates (PHAs).^157^

AB-type long-chain polyesters can also be obtained by ROP of large ring-size lactones.^158−160^ Pentadecalactone is the most studied large ring lactone used for ROP. It is used in the fragrance industry and produced in a five-step synthesis industrially from cyclododecanone.^161^ This route comprises (i) the addition of allylic alcohol, followed by (ii) a ring-closure, (iii) oxidation with H_2_O_2_, (iv) decomposition of the hydroperoxide to an ester, and finally (v) hydrogenation. Though ROP is a chain-growth-type reaction, extensive transesterification can occur during the entropy-driven ROP of large rings with both enzymes and synthetic small-molecule catalysts. This results in product microstructures similar to those from traditional step-growth polyesterification in terms of molecular weight distributions (*Đ* ∼ 2) and random monomer sequences in case of ring-opening copolymerizations of several lactones.^162^ Given the similarities of these linear AB-type long-chain polyesters to A_2_B_2_ polyesters and the effort required for the synthesis of large ring lactones,^163,164^ their utility as monomers compared to long-chain dicarboxylates and diols appears limited.

#### 2.2.2 Long-Chain Polycondensates

**Figure 13:**
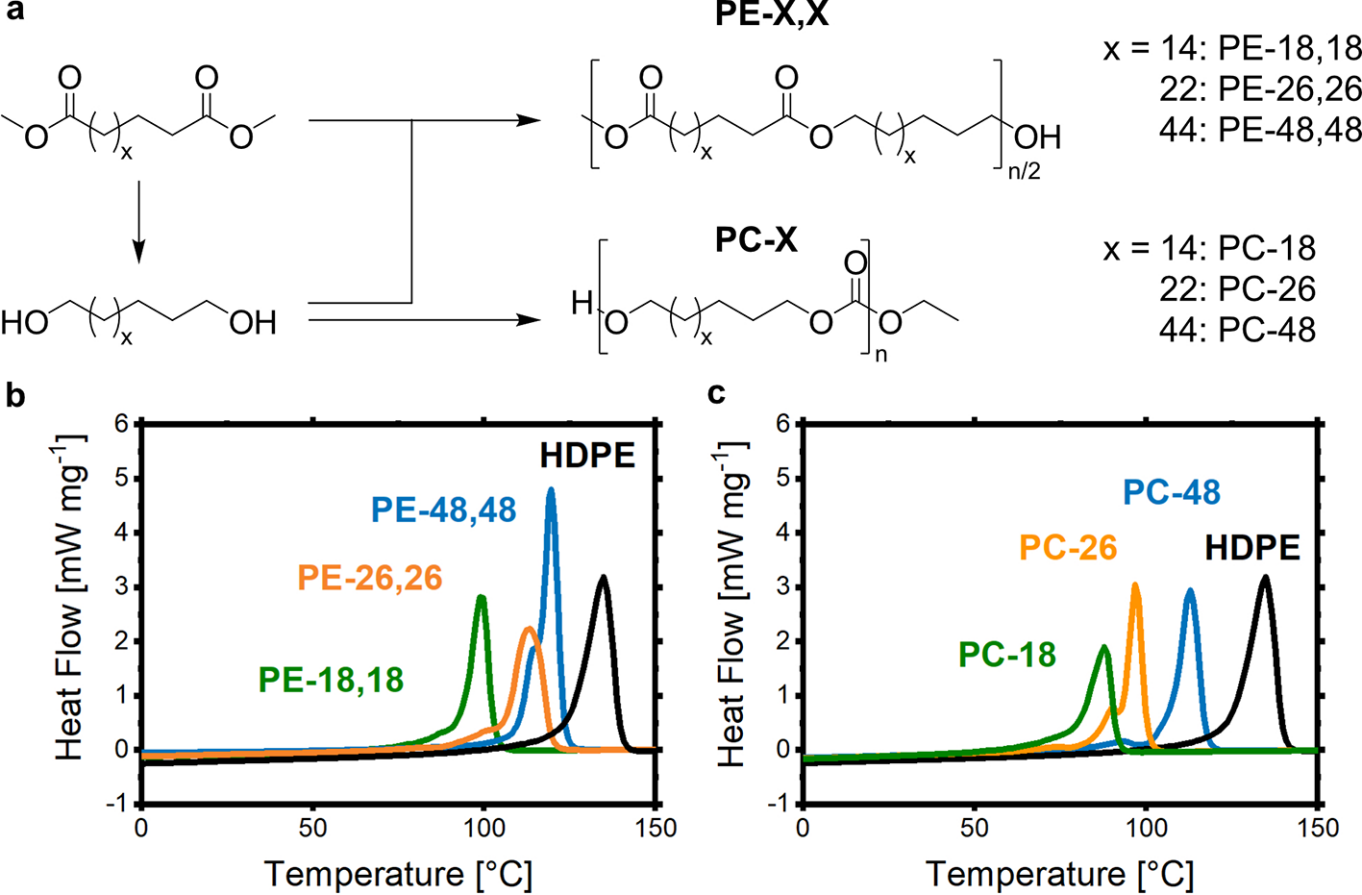
(a) Polymer synthesis of polyesters and polycarbonates via polycondensation. (b) Comparison of differential scanning calorimetry (DSC) traces of polyesters with different chain lengths (C_18_, C_26_, C_48_) between functional groups. (c) Comparison of DSC traces of polycarbonates with different chain lengths (C_18_, C_26_, C_48_) between functional groups.^150,168,172,173^

Polycondensation of the aforementioned long-chain monomers can yield linear aliphatic chains with different in-chain functional groups, polyesters being a prominent example (Figure 13). In these step-growth reactions, high functional group conversions and sufficiently pure monomers are a prerequisite for achieving reasonable molecular weights. In polymerization protocols employing a long-chain dicarboxylic acid or ester in combination with a short-chain diol, the latter is used in excess and removed during the polycondensation along with the more volatile condensate (alcohol or water) liberated from esterification (e.g., final conditions 200 °C, vacuum of 0.1 mbar).^165−167^ By contrast, nonvolatile diols need to be employed in an exact stoichiometric ratio in the initial reaction mixture. On the other hand, the removal of the volatile condensates is less demanding compared to the removal of the excess short-chain diol.^64,168,169^

**Figure 14:**
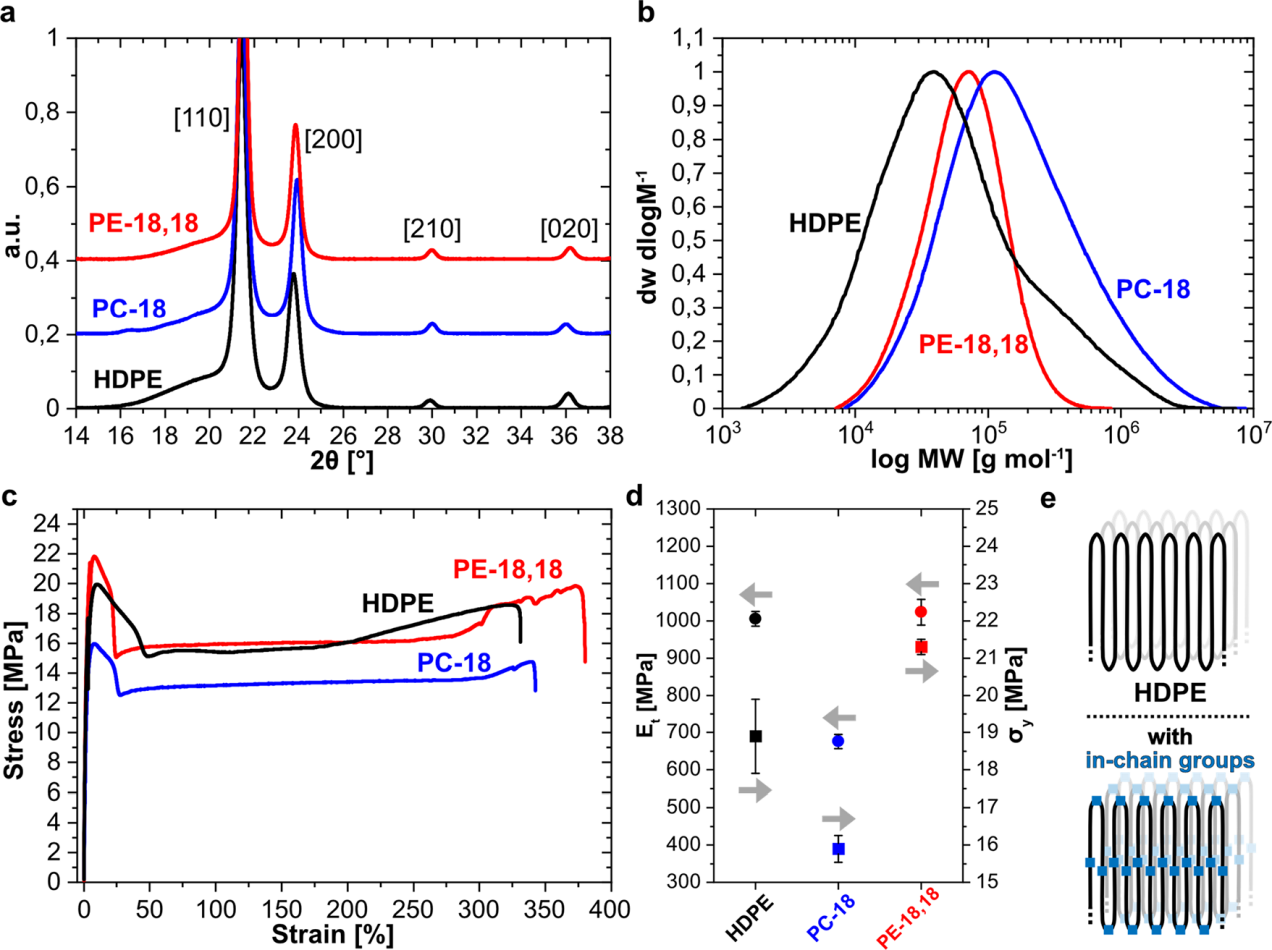
(a) WAXS of PC-18, PE-18,18, and HDPE, reflexes correspond to the orthorhombic unit cell. (b) SEC traces of PE-18,18, PC-18 in comparison to commercial HDPE. (c) Stress–strain curves of PE-18,18, PC-18, and HDPE. (d) Comparison of Young’s moduli and stress at yield values for PE-18,18, PC-18, and HDPE. (e) Schematic representation of the solid-state structure of HDPE (top) and PE-like polymer (bottom) crystallites (Adapted with permission from *Nature***2021**, 590, 423–437. Copyright © 2021, Springer Nature).^168^

Notwithstanding, both approaches can yield polyesters with molecular weights in the range of typical commercial polyethylenes, as illustrated in Figure 14b for polyester-18,18 vs an injection molding grade HDPE. The low density of in-chain ester groups does not disturb crystallization of the chains in an orthorhombic, HDPE-like crystal structure (Figure 14a). Consequently, the modulus, ductility, and toughness of injection-molded specimens are similar to the HDPE reference material (Figure 14c,d).^168^ While the ester groups are preferentially located in the amorphous phase, they can also be incorporated into the polyethylene crystal (Figure 14e).^170,171^ This goes along with an energy penalty, which results in a reduced melting point compared to linear polyethylene, the extent depending on the ester group density (e.g., *T*_m_’s of PE-18,18: 99 °C; PE-26,26: 113 °C; PE-48,48: 120 °C vs HDPE: 135 °C; Figure 13b).^64^

**Figure 15:**
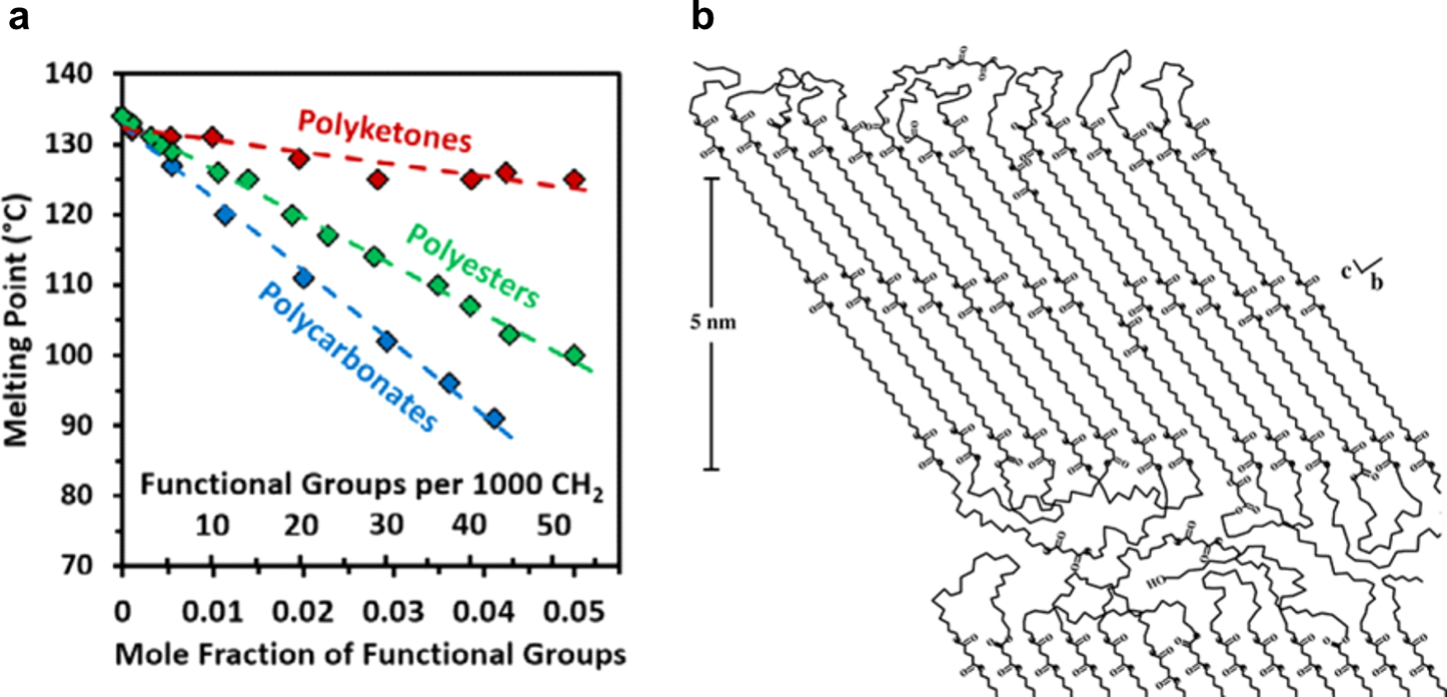
(a) Melting points of randomly long-spaced polyketones (red),^63^ polyesters (green),^175^ and polycarbonates (blue)^176^ vs their density of functional groups (reprinted with permission from *ACS Macro Lett*. **2015**, 4, 704–707. Copyright © 2015, American Chemical Society). (b) Molecular and supramolecular structure of PE-22,4 according to SAXS and NMR spectroscopy (reprinted with permission from *Macromolecules***2007**, 40, 8714–8725. Copyright © 2007, American Chemical Society).^170^

Polyesters from A_2_ + B_2_ polycondensation feature precise methylene spacings between the ester groups as given by the choice of monomers. This enables the formation of layers of ester groups in the crystal, subject to dipole interactions between adjacent segments (Figure 15b). For energetically favorable combinations of methylene spacings, for example, combination of even-numbered dicarboxylates with even-number diols, the dipoles of carbonyl groups in adjacent chain segments in the crystal align in opposite directions, effectively canceling the local polarization. The resulting favorable interactions in part alleviate the energy penalty caused by inclusion of ester groups into the crystalline lattice. This compensation is then reflected in an increased melting point compared to a random arrangement of the functional groups or less suitable combinations of methylene spacings. Consequently, pronounced odd–even effects are observed in A_2_B_2_ polyesters.^64^

Also, long-chain polyesters from mixtures of dicarboxylate monomers of variable length can adopt polyethylene-like structures. Odd–even effects are absent in these polyesters as the irregular spacing of ester groups in the chain hinders favorable dipole alignment, and their melting points are similar to those of the “mismatched” single-length monomer polyesters from odd-numbered dicarboxylates. The *T*_m_ of these “multiplechain length” polyesters increase with a higher center of the monomer-chain length distribution, regardless of the center of the distribution being even or odd. Such polyesters from dicarboxylate mixtures are also of interest as the latter can be potentially sourced from low-value biomass or plastic waste (section 3.2.2).^174^

For a combination of an (even-numbered) long-chain dicarboxylate, with the particularly short C_2_ ethylene glycol, relatively high melting points are observed which may be due to the entire ethylene glycol monomer unit acting as single defect in the crystal (*T*_m_ PE-2,18: 96 °C; *T*_m_ PE-3,18: 82 °C; *T*_m_ PE-4,18: 86 °C; *T*_m_ PE-18,18: 99 °C).^167^ At the same time, ethylene glycol is available on a large scale and at lower cost than long-chain diols, and an increased density of ester bonds leads to further desirable properties, such as an increased environmental degradability (section 3.1).^165,167,174^

The relationship between randomly placed ester, carbonate, and keto group density on polymer properties has been mapped out using hydrogenated model polymers from ADMET. This enables a convenient variation of the functional group density via the ratio of a functional group-containing and a pure hydrocarbon α,ω-diene comonomer (Figure 15a).^63,171,175,176^ A decrease in polymer *T*_m_’s with increasing ester, carbonate, and keto density and with decreasing dipole moment of the functional group from keto to ester to carbonate is instructive.

Studies on copolymers from varying ratios of pentadecalactone (PDL) and caprolactone (CL) revealed further correlations with the crystal structure of the polyesters. Despite a lowering of the melting point and enthalpy of fusion with an increased random incorporation of ester groups (via a higher ratio of CL), the crystallinity of the polymers and also the crystal thickness remained constant over a range of compositions. However, the increasingly irregular stacking of the ester groups resulted in an increased mobility of the chains in the crystallites and lower energy barriers for their deformation, reflected by a significant lowering of the stress at yield for a polymer with equal molar ratios of PDL and CL.^162^

Analogous to PE, the crystallinity of the PE-type polymers can be influenced by the introduction of side chains. Duchateau et al. attached C_5_– or C_6_–OH chains via radical thiol–ene chemistry onto unsaturated macrolactones. The polymerization of these branched monomers yielded an LLDPE-type material with depressed crystallinity and melting points and LLDPE-like mechanical properties.^177^

Barrier properties against water or gases such as oxygen are relevant among others in food packaging applications. The water and gas barrier properties strongly depend on the hydrophobicity and the crystallinity of the material, respectively. Consequently, the gas or oxygen barrier properties of crystalline polyesters like PE-2,16 are favorably similar to HDPE, whereas the water barrier is significantly lower. However, the water barrier is still significantly improved compared to poly(butylene adipate terephthalate), an important commercial biodegradable polyester.^165,178^ While the hydrophobicity of PE is beneficial for barrier properties, it also leads to a low compatibility and thus low adhesion with most other materials. The in-chain ester groups slightly increase the surface free energy of the material,^167^ enabling the adhesion of hydrophilic ink after printing on PE-18,18 films compared to HDPE films. This effect can be enhanced by the introduction of small amounts of ionic groups.^179^

In addition to the polyesters outlined, long-chain polycondensates with numerous other in-chain functional groups have been studied, often motivated by the possibility for deconstruction in recycling or biodegradation.

During the polycondensation of long-chain diols to polycarbonates, diethyl carbonate as a reagent can increase the reactivity of end groups in the critical late stages of the reaction by release of CO_2_ and ethylene to more reactive −OH groups, which has proved beneficial to building up molecular weight. So obtained polycarbonate-18 (*M*_n_ = 90 kg mol^–1^, *M*_w_ = 300 kg mol^–1^) exhibits a PE-like solid-state structure and mechanical properties (Young’s modulus, tensile strength, and ductility) comparable to commercial HDPE (Figure 14). The inclusion of the carbonate groups into PE crystallites goes along with a larger energy penalty compared to polyesters (Figure 15a), which results in somewhat lower melting points compared to polyesters (cf. PC-18: 88 °C vs PE-18,18: 99 °C).^168,176^ Notwithstanding, with sufficiently long methylene sequences also objects from aliphatic polycarbonate material can be made that are not distorted at boiling water temperatures (PC-48: *T*_m_ 113 °C).^168^

Long-chain aliphatic polyamides (PAs) have also been extensively studied; the motivation for these studies was the lower water uptake, higher dimensional stability, and lower melting points of these PAs compared to traditional short-chain polyamides like Nylon-6,6 (*T*_m_ PA-6,6: 265 °C,^180^*T*_m_ PA-18,18: 163 °C^181^). However, hydrogen bonding between the amide groups dominates their properties, and only for very low amide group densities a polyethylene-like structure emerges.^64,149,182,183^

Numerous hydrolytically labile groups have been investigated as part of polymers with long methylene sequences between these functional groups. However, the size and conformational preferences of the explored acetal,^176^ orthoester,^184^ Vitamin C,^185^ and numerous phosphorus-containing groups (H-phosphonates,^186,187^ phenylphosphonates,^186,187^ phosphoesters,^188−190^ or pyrophosphates^191^) impair the formation of a typical orthorhombic polyethylene crystal structure and in this sense compromise a “polyethylene-type” nature of these polymers unless very long spacers (>ca. C_40_) between the functional groups are employed. Apart from a few notable exceptions,^186,188^ mostly brittle materials were reported which may in part also be due to the practical difficulties in determining the tensile properties of rapidly hydrolyzing materials. Much of the understanding of these polymers was enabled by ADMET polymerization of corresponding monomers, developed by Wurm et al.^185,188−191^ However, long-chain poly(H-phosphonates) could also be obtained by straightforward base-catalyzed polycondensation of (ultra)long-chain diols with dimethylphosphonate,^186^ and polyacetals were generated by “acetal metathesis”^192^ from long-chain diacetals.^176,193^ Notably, as blend components such long-chain polymers can be incorporated into a polyethylene-like morphology, likely by cocrystallization, and enhance hydrolytic degradability (Section 3.1).

## 3 Deconstruction

Deconstruction of PE-type materials can proceed via predetermined breaking points incorporated during its chain-growth or step-growth synthesis, as outlined in the previous sections. This enables a closed-loop chemical recycling to the monomers and, in principle, can alleviate the problematic persistence of mismanaged plastic waste by enabling environmental deconstruction through biodegradation (cf. section 3.1). When such breaking points are not present, waste PE may also be recycled by reaction sequences, often starting with dehydrogenation or oxidation (cf. section 3.2).

### 3.1 Via Predetermined Breaking Points

**Figure 16:**
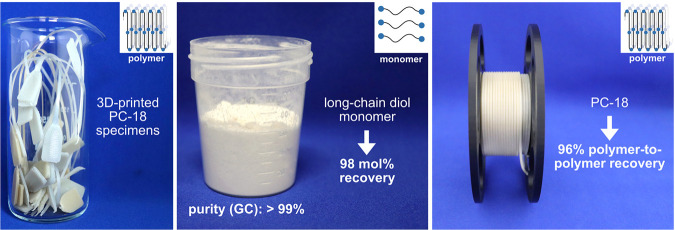
Chemical recycling of PC-18 (Adapted with permission from *Nature***2021**, 590, 423–437. Copyright © 2021, Springer Nature).^168^

Deconstruction to their monomer building blocks via solvolysis was demonstrated for the PE-type polymers PE-18,18 or PC-18 (Figure 13) with methanol or ethanol at 120 to 150 °C, optionally accelerated by KOH as catalyst. The C_18_ diester and C_18_ diol monomers could be recovered in virtually quantitative yield and high purity from mixtures of the respective polymers with polypropylene and HDPE, also separating additives like dyes or reinforcing fibers. Recycled polymer generated from the recovered monomers possesses the same mechanical properties as the virgin polymer, demonstrating the feasibility of a closed-loop recycling of HDPE-like materials (Figure 16).^168^

The similar physical properties such as melting temperature and solubility of the long-chain diol and long-chain diester lead to crystallization of a uniform monomer mixture during recycling of the polyester. The stoichiometric monomer mixture recovered is suitable directly for further polymerization. However, a straightforward separation of the two monomers, if desired, appears challenging.^168^ This could be overcome by short–long chain polyesters, like PE-2,18, whose monomers substantially differ in their physical properties. The isolation of these monomers after solvolysis can be achieved by selective crystallization of the long-chain diacid, and it is further aided by the volatility of the ethylene glycol component.^167^

The depolymerization and repolymerization of PE-type polyesters was found to be robust, as functional groups such as ionic groups^179^ and thioether groups^194^ or furan-containing monomers^195^ did not interfere with the depolymerization process. Even mixtures of diacids could be recovered upon depolymerization in the same ratio as used in the initial polycondensation.^174^ Also, telechelics with high molar masses in the range of several kg mol^–1^ could be de- and repolymerized as described above.^102,136^

The industrial feasibility of solvolysis processes is underlined by the industrial methodology developed for chemical recycling of poly(ethylene terephthalate) (PET). Glycolysis, hydrolysis, or methanolysis at 180 to 250 °C yields terephthalic acid or its esters that can serve as monomers for the polycondensation to PET. Polymer produced in this way meets the stringent requirements of, e.g., food contact materials.^196^ Life-cycle assessments (LCAs) show that the carbon footprint and environmental impact of mechanically and chemically recycled PET is significantly lower than that of PET from nonrecycled resources and, also, comparable to the footprint of energy recovery by incineration.^3,197^ The main contributors to the life cycle impacts of chemical recycling of PET are the high energy input for depolymerization caused by the high melting point and glass transition temperature of PET, the shredding to flakes, and the use of sodium hydroxide as catalyst.^197,198^ By comparison, the lower melting point of polyethylene-type materials is reflected in milder conditions required for solvolysis (120 to 150 °C). In fact, polyethylene-like polyesters could be deconstructed completely to pure monomers in the presence of PET, the latter remaining intact at the lower operating temperatures.^167,168,199^

The chemical recycling of PE-type polymers by solvolysis extends to further in-chain functional groups. Johnson and Johnson recently reported recycling of a material with bifunctional silyl ether groups in a polyethylene chain (generated through a ROMP copolymerization followed by hydrogenation) by reaction with an alcohol.^200^

Although not exactly addressing a polyethylene-like material, it is notable that Greiner and Rist also demonstrated the chemical recycling of a long-chain polyamide, PA-6,19, by hydrolysis.^149^ The monomers could be recovered in a quality suitable for repolymerization into a material with mechanical properties akin to the virgin material.

In general terms, an alternative to chemical recycling via solvolysis that also relies on largely thermoneutral reactions is polymer-cyclic monomer equilibria. The entropy-driven deconstruction of polyamide-6 to ε-caprolactam is applied industrially.^201^ Materials that can be recycled via cyclic acetals and in fact possess tensile properties similar to polyethylene were reported by Coates et al.^202,203^ Thermally stable, processable α-disubstituted polybutyrate can also be recycled via a four-membered lactone.^204^ For the polyethylene-type materials reviewed here with low densities of functional groups in the chain and consequently relatively long methylene segments between functional groups, this would translate to the formation of relatively large rings. The temperature dependency of such polymer-cyclic monomer equilibria is generally less favorable, which also complicates the synthesis of large ring monomers (Section 2.2).

In the processing of postconsumer plastic waste streams, sorting errors^205^ and virtually inseparable multilayer multicomponent materials^206^ can lead to mixed material streams, in particular destructive for chlorinated polymers.^207^ Most polymers, even if relatively similar in their chemical structure, are immiscible.^208^ Such contaminations will result in phase separation during the processing of mechanically recycled polymers. This can result in poor mechanical properties, as prominently exemplified by PE and *i*PP mixtures.^209^ Compatibilization by small amounts of block copolymers can alleviate this problem.^210^

Consequently, polyethylene is immiscible with PE-like polyesters such as PE-19,19, PE-23,23,^169^ and PE-15,^211^ as evidenced by observation of the unaltered melting points of the individual constituents by DSC of blends. However, a good compatibility between HDPE or LDPE and PE-15 was demonstrated by Duchateau et al. using scanning electron microscopy, transmission electron microscopy, and X-ray studies. Epitaxial crystallization from the PE-15 lamellae onto existing HDPE and LDPE lamellae resulted in a fractured surface of the domains, indicating a strong adhesion between the polyester and PE domains. Consequently, clear, ductile films could be prepared from blends of LDPE and PE-15 that crystallized in shish-kebab morphology at higher ratios of PE-15.^211^ The mechanical properties of HDPE were not adversely affected by blending with PE-18,18.^168^ This suggests that a conceivable contamination of polyethylene waste streams with long-chain polyesters during mechanical recycling would not be detrimental. A complete separation of blends of HDPE and a PE-like polyester was also feasible via a selective solvolysis of the latter’s in-chain functionalities, as was shown for HDPE and PE-18,18.^168^

In principle, in-chain ester or other hydrolyzable groups in a polyethylene chain can also enable biodegradation. This can be of interest for particular applications that inherently require biodegradability like compostable packaging or mulch films. More universally, it may provide a backstop for plastic waste littered to the natural environment instead of being delivered to its proper end of life receiving environment. This might alleviate the problematic environmental persistency of polyolefins.^19^ Note that while clear standards for compostability exist, there is no definition of what constitutes a material nonpersisting in the environment.

**Figure 17:**
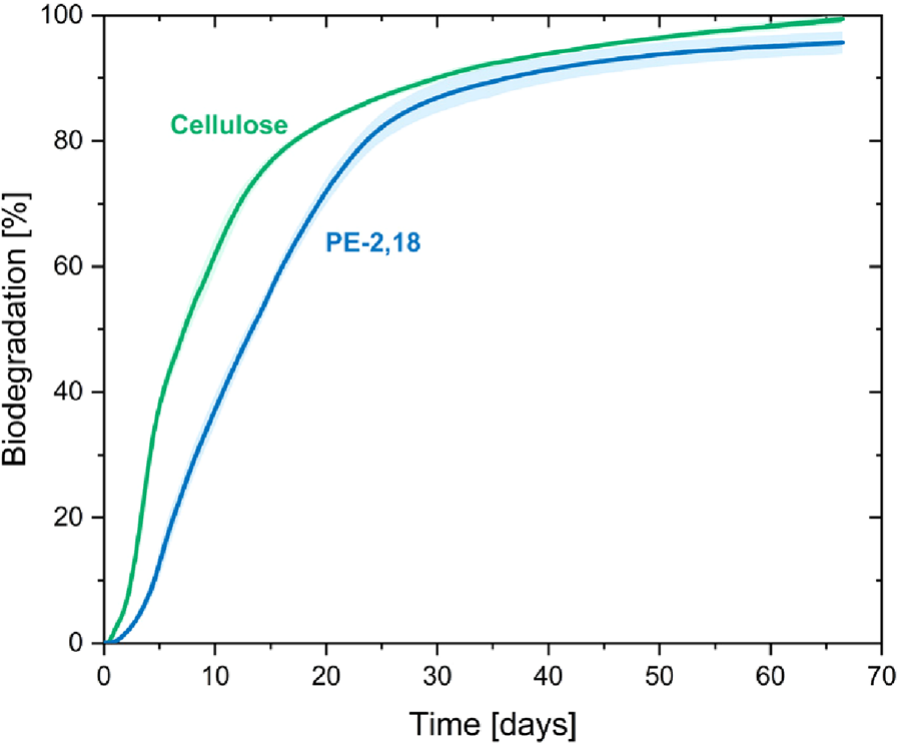
Mineralization of PE-2,18 and cellulose based on CO_2_ evolution under industrial composting conditions at 58 °C following ISO 14855 (reprinted with permission from *Angew. Chem., Int. Ed*. **2023**, 62, e202213438. Copyright © 2023, Wiley-VCH Verlag GmbH & Co. KGaA).^167^

The rate-limiting step of biodegradation is usually the hydrolytic breakdown of the polymer chains by enzymes.^212,213^ Compared to less crystalline polyesters from short-chain monomers, enzymatic or abiotic hydrolysis of HDPE-like materials is expected to be hindered by their crystalline and hydrophobic nature. Indeed, no degradation of PE-15 was observed upon exposure to a buffered lipase solution for 100 days.^214^ PE-18,18 was found to be unaffected by a one-year exposure to dilute hydrochloric acid as observed by monitoring of sample mass and molecular weight via NMR spectroscopy and SEC (size exclusion chromatography).^168^ In contrast, the short–long-type polyester PE-2,18 was found to be degraded by naturally occurring enzymes to the monomers at 37 °C *in vitro* and is fully compostable under industrial composting conditions (Figure 17).^167^ The melting points of polyesters are generally accepted as an indicative factor for degradability.^215,216^ The accelerated degradation of PE-2,18 compared to PE-18,18, despite both polymers exhibiting nearly the same melting point, may be due to the increased ester-bond density or the higher solubility of the monomers of PE-2,18 compared to the water-insoluble C_18_ diol formed upon hydrolysis of PE-18,18.

More hydrolytically labile in-chain groups in long-chain poly-H-phosphonates,^186^ polyorthoesters,^184^ polyphosphoesters,^189^ polyphosphoorthoesters,^217^ and polypyrophosphates^191^ can strongly enhance degradation. For example, poly-H-phosphonate-19 degrades completely to the C_19_ diol and phosphoric acid in 2 days upon exposure to water or moist air. However, the listed functional groups disturb crystallization and hinder adoption of a polyethylene-like solid-state structure of the neat polymer. Notwithstanding, their long-chain structure renders poly-H-phosphonates compatible with PE-18,18 in blends. Upon immersion of these HDPE-like blends in water, the minor hydrolytically labile component degrades completely over the course of months and the liberated phosphoric acid also initiates hydrolysis of the PE-18,18 matrix, resulting in embrittlement and significant molecular weight decrease.^218^

Photolytically cleavable breaking points are another approach toward nonpersistent polymers. Exposure of PE-like keto-polyethylenes from catalytic and free-radical ethylene-CO copolymerization to (simulated) sunlight resulted in a clear onset of degradation, as evidenced by embrittlement, mass loss, and substantial decrease of molecular weight.^32,34,35,60,66^ The amount of keto groups decreases due to Norrish-type chain scission,^219,220^ yet ca. half of the originally present in-chain keto groups remain in those experiments and can promote further degradation. In addition, new carbonyl groups form by accelerated hydrocarbon oxidation in these keto-PEs, which was not observed for an HDPE reference sample within the same period of time.^60^

### 3.2 Post-Use Functionalization

Well over a billion metric tons of chemically inert PE materials have already exceeded their use lifetime, and of the ca. 100 million tons of virgin PE produced annually only a small portion is mechanically recycled.^221^ Thus, waste PE is an abundant feedstock for recycling by chemical methods and also for conversion to other valuable products and chemical intermediates. Substantial efforts for conversion of PE waste into fuel-like hydrocarbon mixtures by hydrogenolytic cracking of the hydrocarbon backbone into smaller fragments have been reported recently.^222−228^ Similarly, alkane cross metathesis between long- and short-chain alkanes can yield fuel or wax-like products.^229,230^ Here, combination of alkane dehydrogenation and olefin metathesis leads to the desired linear alkane mixtures, either by a tandem catalysis with an Ir-catalyzed dehydrogenation and a Re_2_O_7_ olefin metathesis catalyst^229^ or by a single-site, W-catalyst immobilized on silica, which can catalyze both reactions.^230^ All these approaches of hydrogenolytic cracking or alkane metathesis, however, lead to hydrocarbon mixtures, usually suitable for fuel or naphtha applications and therefore limited potential implementation into a circular plastic economy, where a direct reuse of deconstructed intermediates is targeted.

#### 3.2.1 Via Unsaturation

**Figure 18:**
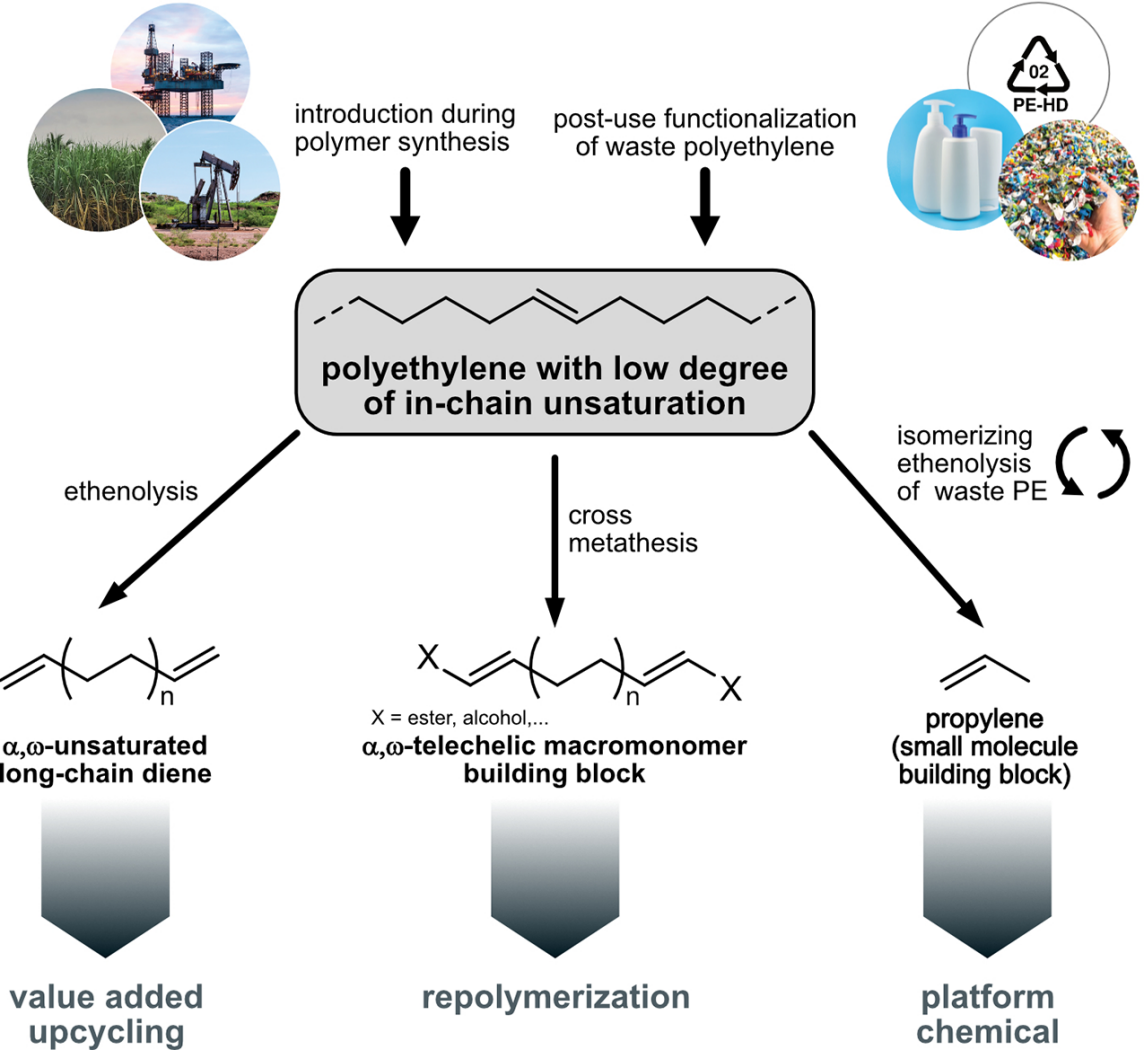
Deconstruction of polyethylene materials via in-chain unsaturation.

**Figure 19:**
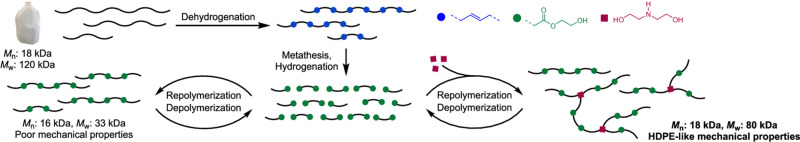
Transforming waste polyethylene to recyclable materials with HDPE-like mechanical properties via consecutive dehydrogenation and cross metathesis (Adapted from *J. Am. Chem. Soc*. **2022**, 144, 51, 23280–23285. Copyright © 2022, American Chemical Society).^237^

On the other hand, the introduction of in-chain double bonds by dehydrogenation can give a valuable platform for more selective chemical deconstruction of waste PE. The obtained in-chain double bonds in these polymers obtained by dehydrogenation of PE chains or also from previous introduction during polymer synthesis (chapter 2.1) can be used as a platform to further chemically modify and eventually deconstruct the polymer (Figure 18). The latter can be achieved by ethenolysis to α,ω-divinyl telechelics^104,105,113,126,231−234^ or cross metathesis with various functional molecules to form long-chain α,ω-telechelic molecules, for example, long-chain diesters or diols.^98,102,110,113,118,235,236^ These can be used as (macro)monomers for polyester or polycarbonate synthesis, thus possibly reintroducing PE waste to a circular economy. For example, Boisson and co-workers synthesized long-chain α,ω-carboxylic acids by oxidation with KMnO_4_^98^ and long-chain α,ω-diols by metathetical depolymerization^110^ from ethylene–butadiene copolymers. Coates et al. applied these concepts to unsaturated propylene-butadiene copolymers, which were cleaved via cross metathesis with acrylates and subsequently repolymerized by polycondensation. The so-obtained ester-linked polypropylene showed properties similar to LLDPE and could be recycled chemically.^102^ Further, the cross metathesis of unsaturated polyethylenes obtained from dehydrogenation^237^ or “blocked” olefins^118^ with acrylates could yield long-chain ester monomers, which were repolymerized by polycondensation to HDPE-like materials (Figure 19).^118,237^

The postuse dehydrogenation of PE chains is usually carried out using Ir-based pincer-type catalysts^229,237−245^ which can lead to a random introduction of unsaturation of up to 3.2 mol %.^241^ These catalysts operate at ca. 200 °C, which facilitates the conversion of the challenging polymeric substrate. Polyethylene is hardly soluble in any solvent at ambient conditions, which can be overcome by high temperatures, above the melting point of polyethylene. The length of the obtained α,ω-telechelic segments after ethenolysis or cross metathesis can be controlled by the amount of unsaturation/dehydrogenation, and segment lengths between approximately 1 and 4 kg mol^–1^ have been reported.^237^ As an alternative to direct catalytic dehydrogenation, the reaction of HDPE with Br_2_ and subsequent elimination of HBr to unsaturated PE chains was reported. Ethenolysis of these unsaturated PE chains resulted in α,ω-divinyl-oligomers with a length of 0.7–0.9 kg mol^–1^.^232^

**Figure 20:**
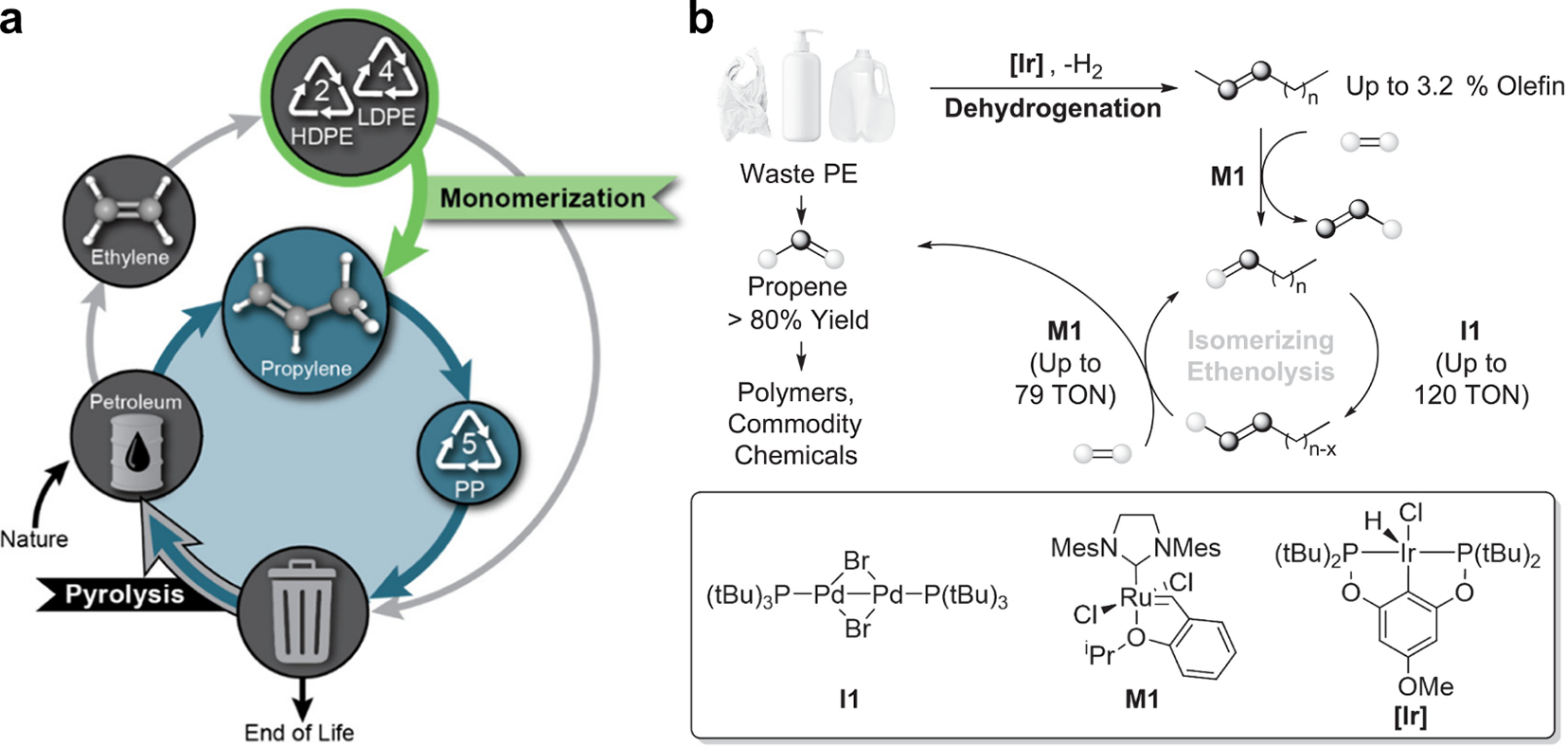
(a) Concept of transforming waste polyethylene to propylene as a valuable chemical building block (Reprinted with permission from *J. Am. Chem. Soc*. **2022**, 144, 18526–18531. Copyright © 2022, American Chemical Society).^240^ (b) Converting waste polyethylene to propylene by dehydrogenation and subsequent tandem isomerizing ethenolysis (Reprinted with permission from *Science***2022**, 377, 1561–1566. Copyright © 2022, The American Association for the Advancement of Science).^241^

The even further deconstruction of PE waste to small platform molecules like ethylene or propylene could enable complete circularity without limiting the products to α,ω-telechelic macromonomers for polymer synthesis only. The chemical recycling of PE back to its ethylene monomer suffers from high energy demands and low monomer selectivity (under 10% effective yields as ethylene).^4,17^ Recently, Hartwig^241^ and Scott and Guironnet^240,246^ independently reported the breakdown of waste PE to propylene in highly efficient yields of up to 80%,^241^ by using a combination of Ir-catalyzed dehydrogenation and subsequent tandem-catalytic isomerizing ethenolysis with Pd-isomerization and Hoveyda-Grubbs^241^/Ultracat^240^ metathesis catalysts (Figure 20). The driving force for this process, which comprises largely energetically neutral conversions, is the utilization of ethylene as a reagent, which also is the origin of two-thirds of the carbon atoms of the formed propylene molecules. Challenges to overcome are the limited temperature stability, and consequently productivity, of the isomerization and (particularly) the soluble, molecular metathesis catalyst (ca. 80 turnovers), with the reaction temperature being dictated by the need to solubilize the polyethylene substrate rather than optimum conditions of the catalysts. To this end, heterogeneous catalysts are potentially more viable in terms of applicability.^240^

#### 3.2.2 Oxidation

**Figure 21:**
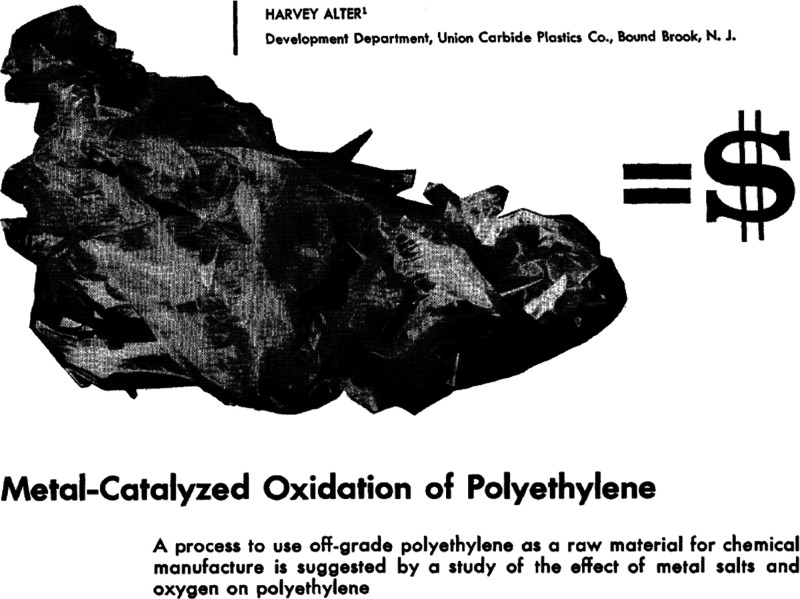
Header of H. Alter’s 1960 article proposing waste PE as a valuable resource (reprinted with permission from *Ind*. *Eng*. *Chem*. **1960**, *52*, 121–124. Copyright 2022, American Chemical Society).^250^

**Figure 22:**
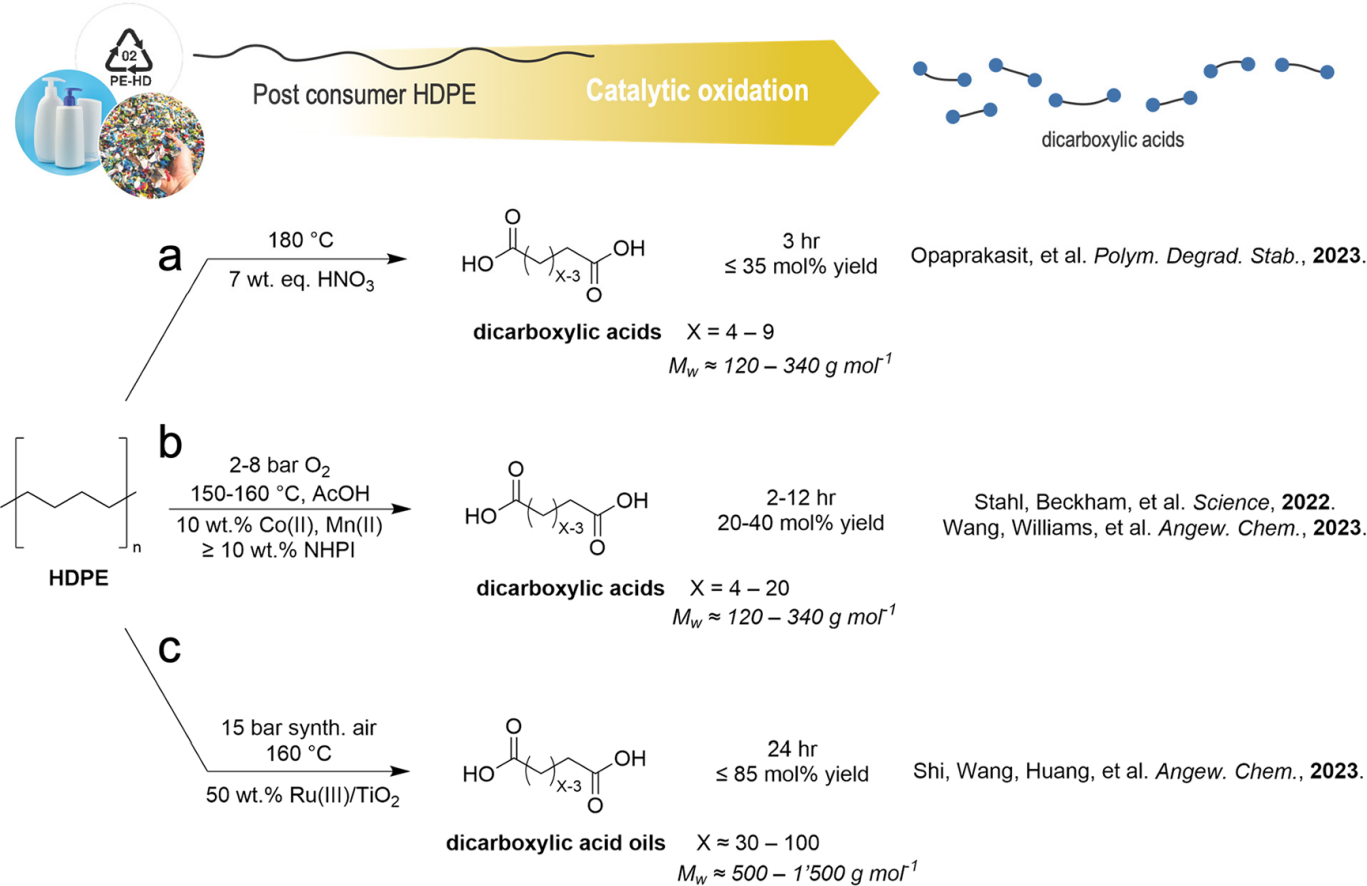
Recent developments and applications of catalytic oxidation processes for the conversion of high-density polyethylene to mixtures of dicarboxylic acids.

A valorization of HDPE waste via oxidation has been suggested already in the very early days of polyolefin technology (Figure 21). Notably, α,ω-dicarboxylic acids (DCAs) can be produced from HDPE by a variety of such oxidative processes (Figure 22). These products may serve as useful intermediates for consumer products, polymers,^174,247^ or as biological feedstocks.^248,249^

Oxidation of PE by hot fuming nitric acid was first developed as a means to isolate crystalline lamellae for the purpose of PE structural elucidation.^251^ Under longer reaction times, PE can be further oxidized, yielding long-chain nitro-carboxylic acids which can be subsequently defunctionalized to give paraffins^252^ or, selectively, DCAs.^253^ The oxidation of PE using nitric acid receives ongoing attention to obtain DCAs as high-value chemicals,^254^ for instance, as monomers to produce polyester acrylates.^255^ With this process, recoveries of C_3_–C_9_ DCAs up to 35 mol % have been achieved after reacting PE with nitric acid (≤7 wt. eq ) for 3 h at 180 °C (Figure 22a).^255,256^

PE can also be oxidized with only gaseous reactants (i.e., nitric oxide/oxygen), also selectively yielding short-chain (C_4_–C_7_) DCAs in recoveries up to 73 wt % after 16 h reaction at 170 °C.^257^ Mild conditions (i.e., room temperature) can also be employed for PE oxidation via sulfonation and subsequent grafting of Fe(III) to PE, enabling Fenton degradation yielding C_4_ DCA and a range of (mono)carboxylic acids.^258^ Later, metal-catalyzed autoxidation of PE, inspired by the industrial process of *p*-xylene oxidation to yield terephthalic acid,^259^ was developed as a preferrable method for the formation of functionalized compounds from polyethylene, especially DCAs.^250^ As such, the state-of-the-art is a cobalt/manganese cocatalyst system with bromide acting as initiator. More recently, *N*-hydroxyimides such as *N*-hydroxyphthalimide (NHPI) have been explored as a less corrosive alternative to bromide.^260^ The utilization of ≥10 wt % NHPI with a cocatalyst system of Co(II) and Mn(II) (added at 10 wt % each compared to PE) under O_2_ pressure and temperatures of 150–160 °C has been shown to produce DCAs in the range of C_4_–C_20_ with 20–40% yields (Figure 22b).^249,261^ Broader distributions of DCAs (up to C_34_) produced without the application of metal catalysts have been observed; however, further oxidized keto-DCAs are produced at the same time.^262^ Furthermore, different conditions (such as oxidation of aqueous PE dispersions)^263^ or catalyst systems (such as Ru(III)/TiO_2_)^264^ have been applied to yield dicarboxylic acid oils (i.e., long dicarboxylate chains with *M*_w_ in the range of 500–2000 g mol^–1^) (Figure 22c). Recent work has also explored the initial feasibility of the deconstruction of HDPE to low molecular weight, oxidized products such as short-chain ketones and carboxylic acids by applying oxidases isolated from wax moth larvae *Galleria mellonella*(265) or from *Rhodococcus spp*. bacteria.^266^

While the oxidation of PE to DCAs has been well-studied, the purification and isolation and the utilization of the resulting products is relatively underexplored to date. Polyesters generated from C_4_ to C_13_ DCA mixtures (from nitric acid oxidation of PE) and 1,4-butandiol or 1,6-hexandiol, respectively, were employed as macrodiols in the production of thermoplastic polyurethane elastomers.^267^ Note this does not require as high a DCA monomer purity as a generation of high molecular weight thermoplastic linear polyesters. Carboxylates from PE oxidation have also been studied as surfactants.^268^

Another key application for multiple chain length DCAs is their direct utilization as biological feedstocks. The bioconversion of PE waste products to PHA was first investigated using PE pyrolysis waxes as feedstocks,^269^ obtained via oxygenation (and ozonation),^270^ to convert low molecular weight PE to paraffins. The shift to transition-metal-catalyzed oxidation yielding a fatty acid mixture, rather than paraffin waxes, was shown to improve their bioconversion efficiency due to improved solubility.^248^ In fact, transition-metal catalysts have long been of interest for the process of paraffin oxidation.^271^ This oxidation strategy has been applied directly to both isolated PE (with DCA yields up to ca. 50 mol %) and PE as part of a simulated mixed-waste stream with other polymers such as polystyrene and PET to produce DCAs.^249,261^ DCAs could then be used as sole carbon source for bioengineered strains of *Pseudomonas putida*, a Gram-negative soil bacterium, for production of PHAs,^249^ or *Aspergillus nidulans*, a filamentous fungi, to produce fungal secondary metabolites, which can be used in medical applications.^261^ In the latter case, as short-chain DCAs (<C_10_) were found to be toxic to select fungal species, they were separated from long-chain DCAs (≥C_10_) using a pH-controlled liquid–liquid extraction.^261^ Notably, recent applications of oxidized PE waste to natural microbial consortia, sampled from plastic recycling plants, demonstrated the possibility to convert these products to wax esters as a valorization product, without the need for specific bioengineering.^272^

It is notable that the chemistry underlying the targeted catalytic oxidation processes has similarities to pro-oxidant additives to PE, used with the aim of catalyzing their inherent oxidation in the environment (so-called “oxo-degradable PEs”). For example, organic salts of Co(III), Mn(II), Fe(III), and Ni(II) have been applied to PE formulations for single-use products.^273^ Other additives used in PE consumer products such as TiO_2_ also inadvertently show photooxidative activity.^274^ While enhanced oxidation of PE results in or accelerates molecular weight decreases and fragmentation, the evidence of subsequent complete biodegradability of such materials remains unproven.^273^ For this and other reasons, “oxo-degradable” plastics have been banned for single-use products by the European Union.^275^ Further assessments of the environmental biodegradability of such materials would require long-term tracking of mineralization of PE-carbon to CO_2_ or assimilation of PE-carbon into microbial biomass, which could be unequivocally verified using stable-isotope labeled materials.^276^

## 4 Conclusions

Compared to unselective breakdown of polyolefin chains by pyrolysis or catalytic cracking at high temperatures to hydrocarbon mixtures, a deconstruction via specific break points can enable a selective chemical recycling to monomers that can be repolymerized to polyolefin-type polymers. Key steps of this deconstruction, as well as synthesis of dedicated monomers and the corresponding construction of polymers already designed for recycling, rely on advanced catalytic methods. Many of the specific catalytic conversions reviewed here can be considered a proof of principle in the sense that further refinements are necessary to take them from the laboratory to a commercial scale. Catalyst performance in terms of activity and particularly stability calls for improvements. Prominently, in the deconstruction of waste polyethylene, elevated reaction temperatures are dictated by the need to operate in the polyolefin melt or in solution. This requires stable catalysts for the reactions of a given deconstruction scheme, like polyethylene dehydrogenation, olefin metathesis, and isomerization. Development of heterogeneous catalysts may provide solutions here. In polyethylene oxidation, achieving sufficient selectivity to dicarboxylic acids (or other products) while maintaining a straightforward approach in terms of oxidant used and number of reaction or purification steps appears to be the key challenge.

That being said, it is encouraging that the general types of reactions employed in the approaches to construction of monomers and polymers and the polymer deconstruction reviewed here, namely, carbonylation, olefin isomerization, olefin cross metathesis, fermentative ω-oxidation, olefin insertion polymerization, ring-opening metathesis polymerization, alkane dehydrogenation or oxidation, polyesterification, and solvolysis are provenly feasible on a (very) large scale.

The utility as materials of the reviewed polymers designed for chemical recyclability and biodegradability has been underlined by demonstration of essential critical features, again on a laboratory or small pilot scale. Long-chain polyesters are melt-processable by injection molding, film extrusion, fiber spinning, or also fused filament printing. They possess desirable tensile properties and barrier properties. Expansion to a larger scale along with in-depth studies and development of specific applications that optionally can comprise recycling is an exciting perspective.

Concerning the environmental deconstruction of polyethylene-type materials via in-chain functional groups, mapping out the impact of ester and other groups on (bio)degradation rates and mechanisms in different environments like soil or marine conditions is imperative.

## Abbreviations

ΔΔ*G*^‡^_alt-nonalt_: difference in Gibbs free activation energies between alternating and nonalternating chain growth
ADMET: acyclic diene metathesis
atm: atmosphere
CL: caprolactone
CTA: chain transfer agent
DCA: dicarboxylic acid
DFT: density functional theory
DSC: differential scanning calorimetry
Cp: cyclopentadienyl
Cp*: 1,2,3,4,5-pentamethylcyclopentadienyl
HDPE: high-density polyethylene
*i*PP: isotactic polypropylene
LCA: life-cycle assessment
LDPE: low-density polyethylene
LLDPE: linear low-density polyethylene
MMAO: modified methylaluminoxane
*M*_n_: number-average molecular mass
*M*_w_: weight-average molecular mass
NHPI: *N*-hydroxyimides such as *N*-hydroxyphthalimide
NMR: nuclear magnetic resonance
OMRP: organometallic-mediated radical polymerization
PA-X,Y: polyamide derived from C_*x*_ diamine and C_*y*_ diacid or diester
PC-X: Polycarbonate derived from C_*x*_ diol
PDL: pentadecalactone
PE: polyethylene
PE-X,Y: polyester derived from C_*x*_ diol and C_*y*_ diacid or diester
PE-Z: linear AB-type polyester with z carbon atoms in the repeat unit
PET: polyethylene terephthalate
PHA: polyhydroxyalkanoate
ROMP: ring-opening metathesis polymerization
ROP: ring opening polymerization
r.t.: room temperature
SAXS: small-angle X-ray scattering
*sc*CO_2_: supercritical CO2
SEC: size exclusion chromatography
*T*_g_: glass transition temperature
*T*_m_: melting temperature
UHMWPE: ultrahigh molecular weight polyethylene
WAXS: wide-angle X-ray scattering
wt %: weight%
